# Focus on cardiac troponin complex: From gene expression to cardiomyopathy

**DOI:** 10.1016/j.gendis.2024.101263

**Published:** 2024-03-11

**Authors:** Rosetta Ragusa, Chiara Caselli

**Affiliations:** a Institute of Clinical Physiology, CNR, via Moruzzi 1, Pisa 56124, Italy; b Fondazione Toscana Gabriele Monasterio, via Moruzzi 1, Pisa 56124, Italy

**Keywords:** Cardiac troponin, Cardiomyopathy, Gene mutation, Post-translational modification, Therapy

## Abstract

The cardiac troponin complex (cTn) is a regulatory component of sarcomere. cTn consists of three subunits: cardiac troponin C (cTnC), which confers Ca^2+^ sensitivity to muscle; cTnI, which inhibits the interaction of cross-bridge of myosin with thin filament during diastole; and cTnT, which has multiple roles in sarcomere, such as promoting the link between the cTnI-cTnC complex and tropomyosin within the thin filament and influencing Ca^2+^ sensitivity of cTn and force development during contraction. Conditions that interfere with interactions within cTn and/or other thin filament proteins can be key factors in the regulation of cardiac contraction. These conditions include alterations in myofilament Ca^2+^ sensitivity, direct changes in cTn function, and triggering downstream events that lead to adverse cardiac remodeling and impairment of heart function. This review describes gene expression and post-translational modifications of cTn as well as the conditions that can adversely affect the delicate balance among the components of cTn, thereby promoting contractile dysfunction.

## Introduction

Cardiac muscle is composed of a contractile apparatus arranged in a pattern that is similar but not identical to the arrangement observed in skeletal muscle. Thin filaments of actin, together with structural and regulatory proteins, interdigitate with thick filaments composed of myosin to form the sarcomere, the functional unit of the contractile apparatus. The “sliding filament theory” proposes that the hydrolysis of adenosine triphosphate (ATP) by the myosin head powers the strong attachment between myosin and actin followed by sliding of the thin filament to generate contraction.^1^

**Figure 1:**
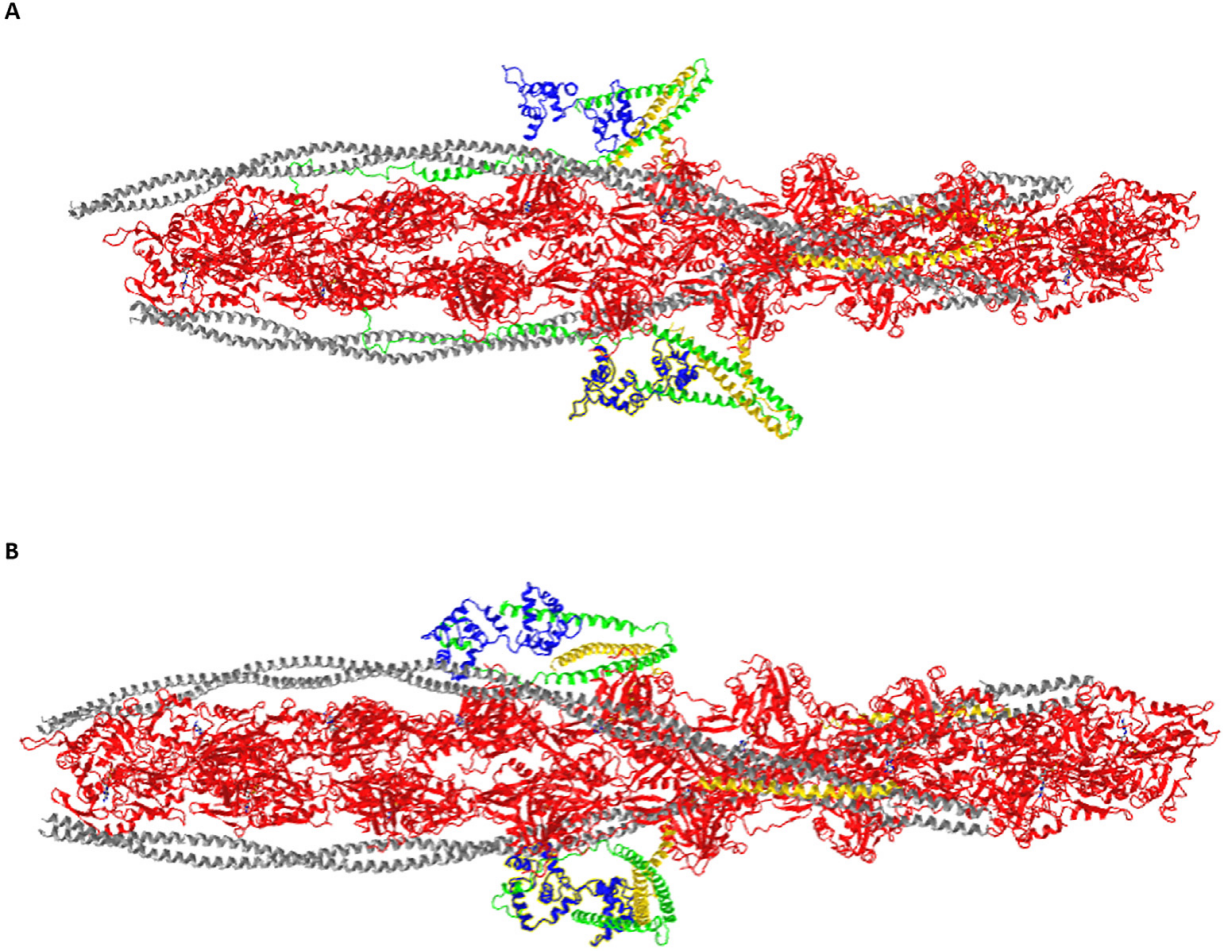
Structure of human cardiac thin filament in the calcium free state **(A)** and in the calcium bound state **(B)**. The monomers of actin are in red and the tropomyosins are in grey; the blue structure represents cTnC, the green element is cTnI, and the yellow protein is cTnT. Figure modified from www.ncbi.nlm.nih.gov/Structure/pdb/6KN8.

In addition to ATP, which provides the energy necessary for muscle contraction, Ca^2+^ is the essential element for sarcomere function. The isometric tension of cardiac muscle fibers is low in the absence of Ca^2+^ but achieves 50% of the maximum tension in the presence of ∼2 × 10^6^ M^−1^ Ca^2+^.^2^^,^^3^ At the beginning of 1900, it was observed in a frog animal model, that the contraction of the isolated heart was blocked in the absence of Ca^2+^.^4^, ^5^, ^6^ Around 1930, it was suggested that, in addition to the extracellular space, the Ca^2+^ is intracellularly stored in the “cortex of the muscle fiber”, and it is quickly released after stimulation, thus regulating muscle contraction.^7^ Later, in 1961, the structure of the sarcoplasmic reticulum was evidenced and the classical “two-state” theory of the contraction and relaxation of the striated muscle was proposed.^8^ Generally, in the classical two-state model of contraction, the increase of cytoplasmic Ca^2+^ triggered by the activation of the action potential induces the release of further Ca^2+^ from the sarcoplasmic reticulum (Ca^2+^-induced Ca^2+^ release). Increased Ca^2+^ levels promote the binding of Ca^2+^ to the “regulatory domain” of the sarcomere, the cardiac troponin complex (cTn), resulting in a cascade of structural changes within the thin filaments,^9^ including the shift of tropomyosin across the surface of the actin filament and the opening of the binding sites for myosin heads.^9^, ^10^, ^11^, ^12^, ^13^ Around 1970, several studies suggested that the contractile cycle was regulated not only by a Ca^2+^-dependent switching mechanism but also by additional steps.^2^^,^^14^, ^15^, ^16^, ^17^, ^18^ In the nineties, the two-step activation/relaxation model was extended to the “three-state model”.^19^ In this model, the states are in dynamic equilibrium and are regulated by the cTn binding to actin, the Ca^2+^ binding to cTn, the myosin binding to actin, the cTn-tropomyosin-actin interaction, and the ATP hydrolysis by myosin (Fig. 1).^20^, ^21^, ^22^ Recently, the three-state model was confirmed by cryogenic-electron microscopy (cryo-EM).^23^, ^24^, ^25^, ^26^

**Figure 2:**
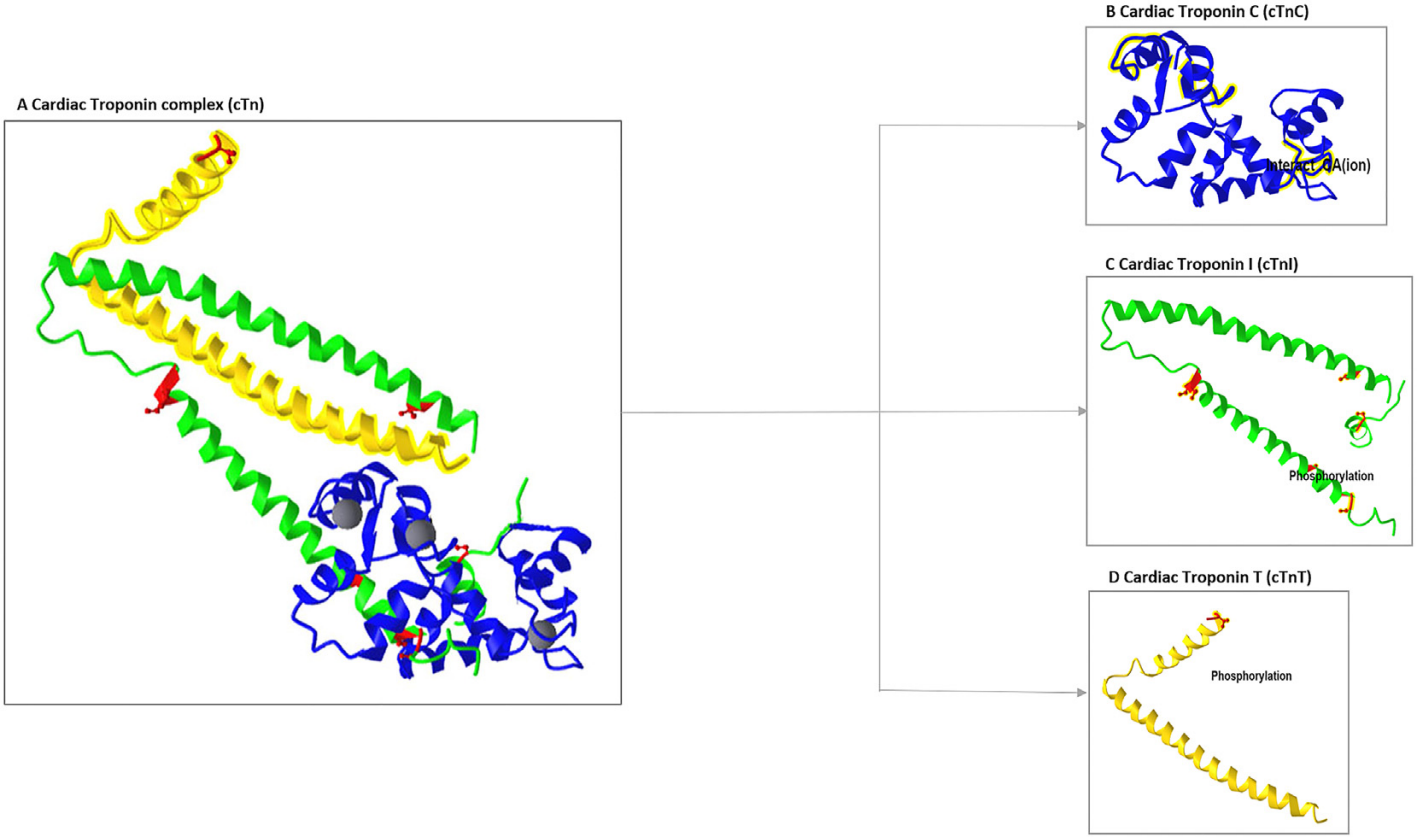
Crystal structure of human cardiac troponin in the calcium saturated. **(A)** Cardiac troponin complex in calcium bound state. **(B)** The cTnC (blue) and its Ca^2+^-binding sites (highlighted in yellow). **(C)** The cTnI (green) and its phosphorylated aa (red). **(D)** The cTnT (yellow) and its phosphorylated aa (red). Figure modified from www.ncbi.nlm.nih.gov/Structure/pdb/1J1D.

In striated skeletal and cardiac muscles, the thin filament is composed of a repeating pattern of actin, Tm, and cTn in 7:1:1 stoichiometry. Comparison between Tn subunits of cardiac muscles with those of striated skeletal muscles is reported in Table S1–4 of Supplementary Material. The cTn has three subunits: the cardiac troponin C (cTnC), the subunit that confers Ca^2+^ sensitivity to muscle; the cTnI, which inhibits the actomyosin cross-bridge formation during diastole; and the cTnT, the tropomyosin (Tm) binding subunit.^27^ A list of genes encoding cTn subunits is reported in Table 1. The X-ray crystallography and the cryo-EM of native or reconstituted thin filaments obtained by cardiac myocytes were essential to establish the structure of the single domains of each cardiac troponin and the orientation of cTn on Tm and actin filament. To date, the cTn crystal structure consists of total TnC and fragments of cTnI and cTnT, covering just 65% of the total cTn lengths (Fig. 2).^26^^,^^28^ The 3D map of the thin filament suggested that cTn could be highly elongated over the seven actin subunits. In this case, the upper four actin subunits are bound by the C-terminal domain of cTnI followed by the cTn “core domain” (cTnC, N-terminal domain of cTnI, and C-terminal domain of cTnT), whilst the bottom three actin subunits of the opposite strand are bound by N-terminal domain of cTnT.^26^^,^^29^

**Table 1:** Gene encoding cTns isoforms.

GENE/chromosome	RefSeqGene	Isoform(adult/fetal)
**TNNC1** **Chromosome3p21.1**	NG_008963.1	TnC (fetal/adult)
**TNNI3** **Chromosome19q13.42**	NG_007866.2	cTnI (adult)
**TNNI1** **Chromosome1q32.1**	NG_016649.2	ssTnI (fetal)
**TNNT2** **chromosome1q32.1**	NG_007556.1	cTnT1 (fetal)
		cTnT2(fetal)
		cTnT3 (adult)
		cTnT4 (fetal)

Among the cTn subunits, cTnI and cTnT are well-known for their pivotal role as biomarkers of myocardial injury. High circulating levels of cTnI and cTnT are commonly associated with diseased states such as acute myocardial infarction, coronary microembolization,^30^^,^^31^ aortic stenosis,^32^ heart failure (HF), myopericarditis,^33^ and atrial fibrillation, or in healthy subjects, in the condition of prolonged exercise. Moreover, a reduction in cTnI and cTnT circulating levels can reflect the efficacies of cardioprotective and prognostic procedures as remote ischaemic preconditioning.^34^ However, the relationship between cTn and myocardial disease extends beyond the cell damage and release of proteins in the blood. Mutations of cTn genes,^35^^,^^36^ the re-expression of fetal cTn variants after birth,^37^ or chronic post-translational modification (PTM) of cTn proteins,^27^ can modify the Ca^2+^ sensitivity of cTn, affecting the duration of systole/diastole phase and contributing to heart disease, including cardiomyopathies and HF.^38^ In this review, the gene expression and the PTMs of cTn were described together with those conditions able to adversely affect the fine balance among the elements of complex finally promoting heart dysfunction.

## Cardiac troponin C

### Gene structure and expression

**Figure 3:**
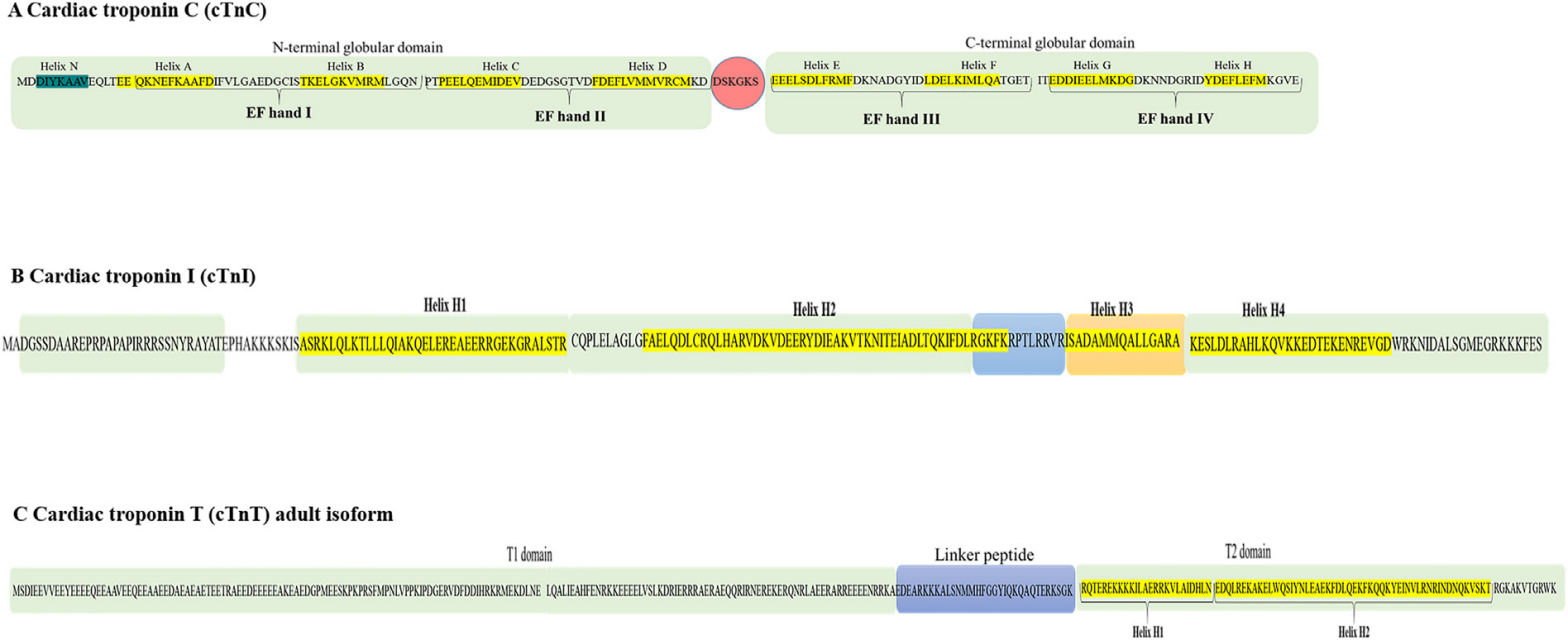
Primary structure of cardiac troponins. **(A)** Cardiac troponin C (cTnC). Schematic representation of cTnC, the sub-unity of cardiac troponin complex (cTn) responsible for binding to Ca^2+^. The rectangular in green represents the N-terminal and the C-terminal domain, whilst the red circle shows the flexible region. The aa involved in the alpha-helix structure are highlighted in yellow (Helix A–H) and dark green (Helix N). **(B)** Cardiac troponin I (cTnI). Schematic representation of cTnI, the inhibitory sub-unity of cardiac troponin complex (cTn). The rectangular in green represents the N-terminal, the IT arm, and the C-terminal domain, whilst the blue and the orange rectangular show the inhibitory peptide region and the switch peptide region respectively. The aa involved in the alpha-helix structure is highlighted in yellow (Helix H1–H4). The post-translational modifications (phosphorylation) are indicated with a “P” within a circle in correspondence with phosphorylation sites. **(C)** Cardiac troponin T (cTnT). Schematic representation of adult cTnT isoform. The rectangular in green represents the T1 and T2 domains, and the blue rectangular shows the linker peptide. The aa involved in the alpha-helix structure is highlighted in yellow (Helix H1 and H2). The post-translational modification (phosphorylation) is indicated with a “P” within a circle in correspondence with the phosphorylation site.

The gene encoding for cTnC is located on chromosome 3p21.1 in humans, on chromosome 14, in mice, and on chromosome 16p16 in rats. The TNNC1 gene is organized in six exons and five introns which encode for cTnC,^18^^,^^20^ a protein of 161 amino acids (aa) (18 kDa) (Fig. 3A).^39^^,^^40^ In contrast to cTnI and cTnT, cTnC is the only isoform present in the heart during both fetal and adult life.^39^^,^^40^

### Protein structure and functional domains

**Figure 4:**
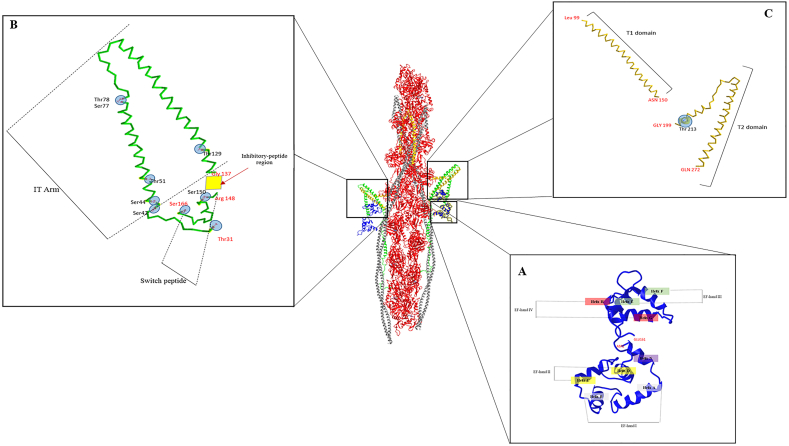
The detailed 3D reconstruction of each component of cardiac troponin complex (cTn). **(A)** The cTnI molecule (from Thr 31 to Ser 166; green) with its resolved structural regions: IT-arm, inhibitory peptide region (yellow rhombus), and switch peptide. The phosphorylated amino acids (post-translational modifications) are reported in circle blue. **(B)** The cTnT (from Leu 99 to Gln 272, in yellow) with its resolved domains (T1 and T2) highlighted. The phosphorylated amino acids (post-translational modifications) are reported in circle blue. **(C)** The cTnC molecule (from Asp 2 to Glu 161, blu 2) with its nine helices and relative EF-hand highlighted.

X-ray crystallography studies showed that the 161 aa of cTnC are arranged in 9 α-helices Helix-N (3–9 aa) and N-lobe helices A–D (aa 14–87) plus C-lobe helices E–H (aa 92–161) (Fig. 4A). The helices A to H create four EF-hands consisting of 30–40-aa motifs from two α-helices (E-helix and F-helix) connected by a loop that forms a unique structure able to bind to divalent cations (as the C-lobe sites are occupied by Mg^2+^ rather than Ca^2+^).^25^^,^^39^ In addition, EF-hand I (16–51 aa) does not bind to divalent cations in cTnC, whilst EF-hand II (52–87 aa) is the regulatory region of cTnC that is highly selective for Ca^2+^ and has a low Ca^2+^ affinity.^41^ In physiological conditions, EF-hand I never binds to Ca^2+^, whilst EF-hand II is linked with Ca^2+^ during systole, thus the EF-hand II is involved in contraction. The EF-hand III (92–127 aa) and IV (128–161 aa) show a high affinity for bivalent ion Ca^2+^/Mg^2+^ and are always occupied by ions.

The N-terminal domain (1–87 aa) is the regulatory element of cTnC and, in contrast, the C-terminal domain is considered the cTnC-structural region (92–161 aa) that keeps cTnC anchored to cTn. When Ca^2+^ concentration (cells range: 10^−7^ to 10^−5^ M) increases in the cytoplasm and binds to the EF-hand II, the cTnC changes conformation, leading to the opening of a hydrophobic patch able to bind to the switch region on cTnI and to induce cTn changes, with consequent sarcomere contraction.^41^

## Cardiac troponin I

### Gene structure and expression

The gene encoding for cTnI is located on chromosome 19q13.42 in humans, on chromosome 7 in mice, and on chromosome 1q12 in rats. The TnI is expressed in the human heart in two age-dependent-isoforms that change during development. Slow skeletal troponin I (ssTnI) is the expression product of the TNNI1 gene (chromosome 1q32.1) and is predominantly synthesized in the slow skeletal muscle but also the heart during embryonic life. The TNNI1 gene, characterized by 9 exons and 8 introns, encodes a protein of 187 aa. ssTnI is completely replaced by cTnI in 9–24 months after birth in the human heart,^42^, ^43^, ^44^ while in an animal model, it was found that TNNI1 gene was expressed up to 15 days after birth.^45^ In humans, the cTnI gene (TNNI3) includes 8 exons and 7 introns and encodes for a flexible protein of 210 aa (24 kDa). It was suggested that the replacement of ssTnI with cTnI during newborn life could be associated with increased responsiveness to the β-adrenergic system.^44^ The Ser 23/24 at the N-terminal domain of cTnI is regulated by protein kinase A (PKA) which is, in turn, activated by the β-adrenergic system,^46^ but the N-terminal domain is missing in ssTnI.

### Epigenetic regulation

In recent years, epigenetic regulation of TnI expression has been studied. Epigenetics mechanisms may fine-tune the gene expression by chromatin remodeling (acetylation/deacetylation and methylation/demethylation of histone) or by silencing the mRNA (long non-coding RNA and microRNA/miRNA). Hence, the role of miRNA-449 in regulating cTnI expression was demonstrated.^47^ Low levels of acetylation of cTnI promoter are associated with a low cTnI expression in elderly mice.^48^ In *in vivo* and *in vitro* studies, high levels of miR-449 could indirectly increase the cTnI levels through the binding and degradation of histone deacetylase 1 mRNA.^26^ In this way, acetylation of the GATA element and recruitment of transcription factor to the TNNI3 promoter region was favored.^49^ On the other hand, non-coding RNA, such as miRNA-208b and miRNA-499, can regulate the expression of “fast skeletal troponins” TNNT3 and TNNI2, troponins of striate skeletal muscle, also in the mouse heart.^50^^,^^51^

### Protein structure and functional domains

cTnI is organized into five domains with different functions (Fig. 3B). The N-terminal domain (2–32 aa) is a finely regulated region characterized by two adjacent serine residues (Ser 23-Ser 24), that can be phosphorylated by PKA or other kinases, under stress conditions and/or exercise, thus affecting the time of Ca^2+^ release from cTnC and, consequently, the sarcomere contraction.^10^^,^^13^

The second domain of cTnI, the IT arm (42–136 aa), is composed of two α-helices, Helix H1 (43–79 aa) and Helix H2 (90–135 aa), linked by a flexible-U-turn sequence.^41^ The IT arm is the structural domain of cTnI able to bind to cTnT by a rigid coil-to-coil interaction^52^ and is a valid anchoring site for the C-terminal domain of cTnC.^10^^,^^13^^,^^53^ The classical model describes that the C- and N-terminal parts of the IT arm are near and parallel to the actin-tropomyosin filament.^54^ The cryo-EM map resolution showed that only the C-terminal part of the IT arm is close to the actin-tropomyosin, whereas the N-terminal end is perpendicular to the long axis of thin filament together with the Helix D of cTnC.^9^^,^^54^^,^^55^

Beyond the IT arm, the C-terminal domain of cTnI is composed of the inhibitory-peptide region (137–148 aa) and the switch peptide (149–164 aa) (Fig. 4B).^53^, ^54^, ^55^, ^56^, ^57^, ^58^ Biochemical studies demonstrated that, in the condition of low Ca^2+^ concentration, the EF-hand II of cTnC is closed and the switch peptide is far from cTnC and tightly bound to actin-Tm.^26^ In this state, the inhibitory peptide stabilizes the Tm on the actin filament in a “blocked position”, preventing the entrance of the myosin head into the thin filament.^26^^,^^59^^,^^60^ In addition to cTnI–Tm interaction, the molecular model of human cardiac thin filament studied by cryo-EM suggested an intimate electrostatic interaction between arginine residues 145, 146, and 148 of TnI inhibitory peptide and Asp 24 and 25 of actin filament.^21^^,^^26^ In the condition of high Ca^2+^ concentration, Ca^2+^ can bind to the EF-hand II of cTnC, opening the hydrophobic patch in the N-terminal domain of cTnC and making possible its interaction with the switch peptide of cTnI.^41^^,^^61^, ^62^, ^63^, ^64^ Thus, the C-terminal domain of cTnI can reduce the regulatory activity of cTn on actin-Tm, facilitating the shift of Tm to a “closed” position and, consequently, the access of myosin heads to actin.^21^^,^^26^ The Tm transition in the “three-state” model of contraction and the exact binding sites of Tm and cTn on actin are still partially unclear.^21^

### Post-translational modifications

PTMs could change the properties of cTn by proteolytic cleavage or by the addition of biochemical groups (phosphorylation or glycosylation mechanisms) to one or more amino acids. Under physiological conditions, PTMs play a key role in numerous biological processes. The main PTMs observed for cTnI is the phosphorylation of functionally relevant amino acid residues, scattered throughout the protein, such as Ser 23/24, tyrosine 26, Ser 42/44, threonine (Thr) 5, Ser 77, and Thr 78/143. Among them, the Ser 23/24 phosphorylation could be considered as a mechanism useful to accelerate the diastolic relaxation period, thus improving cardiac function.^65^^,^^66^ Conversely, other modifications of cTnI, such as Ser 150 phosphorylation, could increase myofilament Ca^2+^ sensitivity and blunt the Ca^2+^ desensitization induced by Ser 23/24 phosphorylation.^67^

The effect of cTnI phosphorylation on cardiac contraction has emerged from biophysics studies in isolated myofibrils.^68^ The phosphorylation of Ser 23/24 is a physiological event activated by the increase of catecholamine circulating levels. Specifically, catecholamines induce the cTnI Ser 23/24 phosphorylation by PKA activation,^69^^,^^70^ thus improving heart performance by the reduction of the heart contraction-relaxation period.^71^ The classical theory of Ser 23/24 phosphorylation suggests that Ser 24 is modified before Ser 23 and that only the bi-phosphorylation reduces the switch peptide's ability to bind to the hydrophobic patch of cTnC, thus inducing the release of Ca^2+^ from EF-hand II in an indirect way.^72^^,^^73^ Even if the bi-phosphorylation of Ser 23/24 mediated by PKA is considered the essential condition required for Ca^2+^ sensitivity reduction of the thin filament, PKD-mediated phosphorylation of only Ser 24 can largely reproduce the PKA effects.^19^

Besides PKA and PKD, Ser 23/24 can be phosphorylated by other kinases, such as PKG and PKC. However, whilst PKA, PKD, and PKG can phosphorylate only Ser 23/24 on cTnI, PKC phosphorylates also other sites, such as Ser 42/44 or Thr 143.^74^ In mice, improvement in cardiac contraction dependent on Ser 23/24 phosphorylation and PTMs of Ser 43/45 or Thr 144 (equivalent to Ser 42/44 and Thr 143 in human) could reduce myofilament Ca^2+^ sensitivity and develop a reduction of pump function.^75^^,^^76^ Molecular mechanisms and conformational changes activated by the phosphorylation of these sites have not yet been adequately studied and there is still a lot to understand about their potential effect in heart disease.

In addition to phosphorylation, cTnI could be subject to other PTMs. Elevation of glucose modifies various proteins by O-linked β-N-acetylglucosamine glycosylation of serine or threonine residue. Specific O-linked β-N-acetylglucosamine glycosylation at Ser 150 of cTnI was found in cardiac trabeculae isolated from mice that could modify the myofilament Ca^2+^ sensitivity.^7^

## Cardiac troponin T

### Gene structure and expression

The gene encoding for cTnT is located on chromosome 1q32.1 in humans, on chromosome 1 in mice, and on chromosome 13q13 in rats. The cTnT is recognized as the structural component of cTn that firmly fixes the complex to the thin filament of the sarcomere, via tropomyosin. Moreover, cTnT has also an active role in the “three-state model” of muscle contraction, interacting with TnC, TnI, and tropomyosin, and, in particular, regulating the strong cross-bridge attachment to actin to generate force.^25^^,^^54^^,^^76^ The cTnT is encoded by the TNNT2 gene and is composed of 17 exons and 16 introns. During the development of the human heart, alternative splicing of exons 4 and 5 generates up to four isoforms: cTnT1 (with exons 4 and 5), cTnT2 (with only exon 5), cTnT3 (with only exon 4), and cTnT4 (without both exons).^37^ The fetal heart predominantly expresses cTnT1 (298 aa) and, to a lesser extent, cTnT2 (293 aa) and cTnT4 (283 aa). After birth, a regulated switch occurs from the embryonic isoforms to the cTnT3 (288 aa), the only expressed isoform in the adult heart.^37^ Besides the canonical isoforms, cTnT1, cTnT2, cTnT3, and cTnT4, generated by the variants 5, 8, 2–7, and 4, respectively, further non-canonical cTnT isoforms, cTnT10, cTnT11, and cTnT12, are produced by other variants (Table 2). However, the role of non-canonical isoforms of cTnT within sarcomere is still unknown.

**Table 2:** Canonical and non-canonical isoforms of human cardiac troponin T (cTnT).

	Canonical/non Canonical	N. GenBank access
cTnT1	Canonical	NM_001276345.1
cTnT2	Canonical	A0A0A0MRJ4
cTnT3	Canonical	NM_001001430.2
cTnT4	Canonical	NM_001001432.2
cTnT10, 11,12	non Canonical	NM_001276346.1 NM_001001431.2

### Protein structure and functional domains

Isoforms of cTnT are organized in three domains (Fig. 3C): the N-terminal region, the flexible linker, and the C-terminal region. Within the N-terminal region, the T1 domain (1–168 aa for cTnT3) is a highly flexible domain that binds cTnT to Tm.^37^^,^^77^ Cryo-EM studies showed that a small part of the N-terminal region of cTnT interacts with the N-terminal end of Tm located at the same contractile subunit (7 actins, 1 cTn, and 1 Tm).^26^^,^^29^ The rest of the T1 domain crosses the tropomyosin N-/C-terminal overlapping domain and then extends over the C-terminal part of the adjacent Tm molecule.^26^^,^^29^ Whitin the cTn core,^77^ the T2 domain (201–288 aa for cTnT3) is characterized by two α-helices (Helix H1 and Helix H2) and by the C terminal peptide (278–288 aa) (Fig. 4C).^77^ Whilst the Helix H2 forms an antiparallel coiled-coil with the Helix H2 to cTnI (IT arm), interacting also with the C-terminal domain of cTnC,^13^^,^^41^ the C-terminal peptide of cTnT (16 aa, commonly unstructured) can bind to the inhibitory-peptide of cTnI, the N-terminal peptides of cTnT in the hinge region, and, less frequently, the TnT Helix 2 and the C-terminal domain of TnC.^13^

cTnT can modulate Ca^2+^ sensitivity. The expression of exon 5 makes the N-terminal region of cTnT1 and cTnT2 highly acidic and negatively charged at physiological pH. Moreover, compared with human cTnT4 with an isoelectric point = 5.2, the human cTnT1 has an isoelectric point = 4.94, showing a higher Ca^2+^ sensitivity.^78^ Embryonic cardiac TnT with more negative charges in the N-terminal region and a lower isoelectric point can improve the Ca^2+^ sensitivity of cTn in an environment with low Ca^2+^ concentration and increase the force of muscle contraction, compared with the adult isoform of cTnT.^77^, ^78^, ^79^ Thus, in the context of development or cardiac disease, cardiac troponin undergoes functional adaptations by isoform switches to respond to an altered cardiac demand. Indeed, fetal isoforms of cTnT are re-expressed in the heart of HF patients, in particular the cTnT4 isoform.^37^^,^^80^ Specifically, the TnT N-terminal charge could alter the affinity to cTnI and Tm and the more acid TnT (isoelectric point = 6.33) could have less affinity for Tm compared with the more basic TnT (isoelectric point = 8.99).^81^

### Post-translational modifications

At the beginning of the 1980s, different research groups found an enzyme indicated as troponin T kinase able to phosphorylate bovine or dog cTnT in a single site (Ser 2) located at the N-terminal domain.^52^^,^^82^^,^^83^ Subsequently, other phosphorylation sites modified by PKC activity have been found in purified cTnT, such as Ser 208, Thr 213, and Ser 285.^59^ Moreover, the number of phosphorylated sites on cTnT increased after prolonged incubation of cTnT with PKC.^74^

In addition to PKC, other kinases such as apoptosis signal-regulating kinase 1, calmodulin-dependent protein kinase II, Rho-A-dependent protein kinase II, and Raf-1 proto-oncogene serine/threonine-protein kinase can phosphorylate “purified” cTnT in Thr 204, Thr 206, Ser 208, Thr 213, Ser 285, and Thr 294, suggesting that PTM of cTnT could be regulated from different pathways.^84^^,^^85^ In *in vitro* studies, purified or recombinant cTnT is exposed to kinases for phosphorylation, and to cTnI, cTnC, and Tm for assembling cTn, and to hook the complex to thin filament.^86^ These phosphorylations avoid the binding of cTnT to Tm but not to cTn, thus reducing the ability of sarcomere contraction. In particular, the phosphorylation of Thr 213 is associated with a reduction of Ca^2+^ sensitivity and maximum tension development by myofilament. Unlike, *in vitro* studies using top-down protein mass spectrometry applied to rat and human heart samples showed that cTnT was predominantly monophosphorylated (Ser 2) in physiological conditions.^42^, ^43^, ^44^, ^45^, ^46^, ^47^, ^48^, ^49^, ^50^, ^51^, ^52^, ^53^, ^54^, ^55^, ^56^, ^57^, ^58^, ^59^, ^60^, ^61^, ^62^, ^63^, ^64^, ^65^, ^66^, ^67^, ^68^, ^69^, ^70^, ^71^, ^72^, ^73^, ^74^, ^75^, ^76^, ^77^, ^78^, ^79^, ^80^, ^81^, ^82^, ^83^, ^84^, ^85^, ^86^, ^87^

## Cardiac troponin in cardiovascular disease

**Figure 5:**
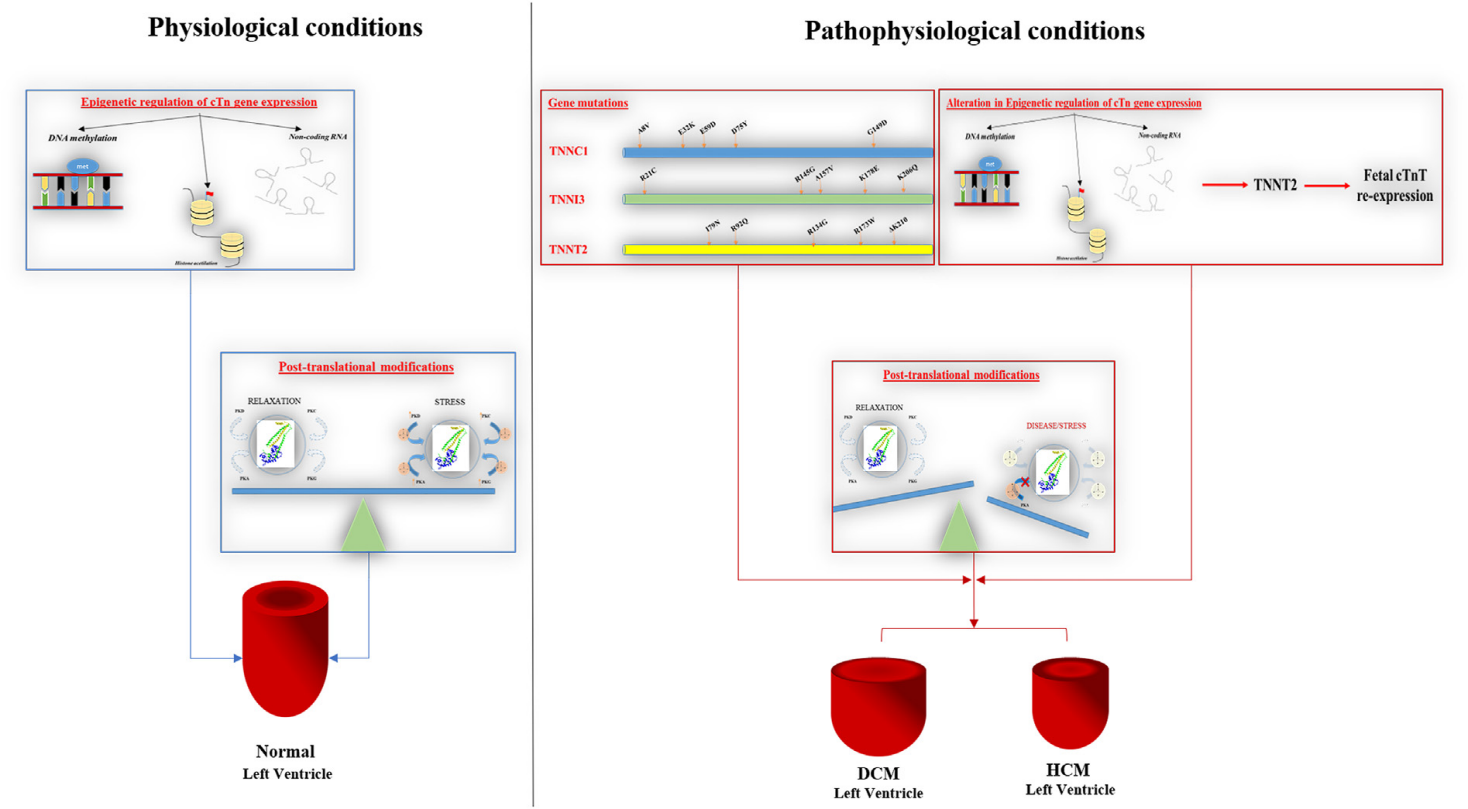
Molecular conditions that can influence the cardiac troponin complex expression and function. **(A)** The action of epigenetic regulation and post-translational modifications in physiological conditions. **(B)** Effects of gene mutation**,** epigenetic de-regulation, and post-translational modifications in pathophysiological conditions.

Considering the key role of cTn in the regulation of cardiac contraction, conditions able to modify cTn gene expression, such as mutations in the cTn genes and re-expression of fetal cTn isoforms, could affect the sarcomere function. These molecular changes might affect cardiomyocytes leading to progressive dysfunction. How specific molecular changes could lead to different disease phenotypes, including dilated cardiomyopathy (DCM), hypertrophic cardiomyopathy (HCM), restrictive cardiomyopathy (RCM), and left ventricular non-compaction cardiomyopathy, is still under investigation. Moreover, besides regulation of gene expression, disruption or chronic activation of PTMs could contribute to the starting and progression of pathological states with long-term consequences that lead to cardiac dysfunction and HF (Fig. 5).

### Gene mutations

Mutations in TNNC1, TNNI3, and TNNT2 have been identified as relatively uncommon causes of cardiomyopathy, such as DCM, HCM, and RCM, when compared with the frequency of mutations in other sarcomere genes,^36^^,^^37^^,^^88^^,^^89^ probably because most of them are not compatible with life. Known mutations into the cTnC-cTnI-cTnT genes were listed in Table 3, Table 4, Table 5, respectively, together with the molecular effect and the clinical condition related to each mutation (as indicated by the American College of Medical Genetics and Genomics classification). The molecular effect was not reported in the tables for those variants identified only by genetic testing and then recorded in the ClinVar database without any pathophysiological verification in experimental models. Conversely, mutations whose molecular effects have been studied at the molecular/pathophysiological level by dedicated studies are described in column 2 of each table.

**Table 3:** Cardiac troponin C (cTnC) pathogenic/likely pathogenic mutations.

Protein change	Molecular effects	Condition(s)	ACMG classifications	Reference
G159D	G159D-cTnC mutant reduced the binding affinity of cTnC on cTnI.	DCM	*Likely pathogenic*	142
C84Y	C84Y-cTnC mutant induced alterations in protein secondary structure that modifies dynamic interactions of cTnC with other cardiac thin filament proteins	Familial HCM	*Pathogenic*	143
E56D	–	DCM, Familial HCM	*Likely pathogenic*	https://www.ncbi.nlm.nih.gov/clinvar
V44M	V44M-cTnC increased the interaction with calcium compared to wild-type cTnC.	HCM	*Likely pathogenic*	https://www.ncbi.nlm.nih.gov/clinvar
A31S	A31S-cTnC mutant increased Ca^2+^ sensitivity with no effect on the maximal contractile force generation	Familial HCM	*Pathogenic*	11
A8V	cTnC-A8V mutant increased Ca^2+^ sensitivity and led to diastolic dysfunction.	HCM	*Pathogenic/Likely pathogenic*	42,88
D75Y, E59D, E32K	D75Y, E59D, and E32K-cTnC mutants decreased Ca^2+^ sensitivity of thin filament and were associated with DCM	DCM	*Likely pathogenic*	92, 93, 94

DCM: Dilated Cardiomyopathy; HCM: Hypertrophic Cardiomyopathy.

**Table 4:** Cardiac Troponin I (cTnI) pathogenic/likely pathogenic mutations.

Protein change	Molecular effects	Condition(s)	ACMG classifications	Reference
K206Q	K206Q mutation in the C-terminal region of cTnI influenced the Ca^2+^ sensitivity of thin filaments.	Familial HCM	*Pathogenic*	144
R204H	R204 mutation in the C-terminal region of cTnI influenced the Ca^2+^ sensitivity of thin filaments.	HCM, RCM	*Pathogenic*	144
G203S	G203S mutation in the C-terminal region of cTnI influenced the Ca^2+^ sensitivity of thin filaments.	Familial HCM	*Pathogenic*	144
D196N	D196N mutation in the C-terminal region of cTnI influenced the Ca^2+^ sensitivity of thin filaments.	HCM	*Pathogenic/Likely pathogenic*	144
R192H, R192C	R192H-cTnI and R192C-cTnI decreased the tension developed in the sarcomere.	Familial HCM, RCM	*Pathogenic*	145
D190Y	D190Y-cTnI mutant might affect the interaction of cTnI with the other proteins of thin filaments.	HCM	*Likely pathogenic*	146
R186Q	R186Q mutation in the C-terminal region of cTnI influenced the Ca^2+^ sensitivity of thin filaments.	Familial HCM	*Pathogenic/Likely pathogenic*	145
N185K	N185K-cTnI mutant decreased maximal ATPase activity and decreased calcium sensitivity of actin-myosin S1 ATPase.	DCM	*Pathogenic*	147
ΔK178	ΔK178 mutation in the C-terminal region of cTnI influenced the Ca^2+^ sensitivity of thin filaments.	HCM	*Pathogenic*	145
K178E	The fiber with K178E-cTnI mutation was unable to fully relax contraction in the absence of Ca^2+^.	Familial RCM	*Pathogenic*	99
R170Q	R170Q-cTnI mutation within the actin-binding domain has been presented to cause excessive inhibition in troponin I actomyosin ATPase activity.	HCM, RCM	*Pathogenic*	99
R170W	R170W cTnI variant impaired the communication between thin and thick filament proteins and destabilized thin filaments.	HCM, RCM	*Pathogenic/Likely pathogenic*	53
S166F	S166F-cTnI mutation in the C-terminal region of cTnI influenced the Ca^2+^ sensitivity of thin filaments.	Familial HCM	*Pathogenic/Likely pathogenic*	144
R162P, R162Q, R162W	All mutations reduced the interaction of cTnI with cTnC.	HCM, DCM	*Pathogenic/Likely pathogenic*	144
A157V	A157V-cTnI mutation reduced the interaction of cTnI with cTnC.	Primary familial HCM	*Pathogenic*	144
R145Q	R145Q-cTnI mutant decreased inhibition of ATPase activity when compared with wild-type cTnI.	HCM, DCM	*Pathogenic/Likely pathogenic*	99
R145W	R145W mutation caused a significant increase in Ca^2+^ sensitivity of force development.	HCM	*Pathogenic*	99
R145G	R145G-cTnI mutant was associated with HCM	Familial HCM	*Pathogenic*	93,95,98
L144P	L144P-cTnI mutant altered the inhibitory domain of cTnI.	HCM	*Likely pathogenic*	144
L144Q	L144Q-cTnI mutant altered the inhibitory domain of cTnI.	RCM	*Pathogenic*	144
R141Q	R141Q-cTnI mutant altered the inhibitory domain of cTnI.	HCM	*Pathogenic/Likely pathogenic*	144
D127Y	D127Y-cTnI mutant disrupted the structural integrity of the sarcomere.	Familial RCM	*Likely pathogenic*	148
R21C	R21C-TnI mutants were fibers characterized by an increase in Ca^2+^ sensitivity, force development, and reduction in phosphorylation to Ser 23/24 by PKA when compared to wild-type fibers.	HCM	*Pathogenic*	99
M1V, L135P, R192P, R192L, M201T	–	Primary DCM	*Likely pathogenic*	https://www.ncbi.nlm.nih.gov/clinvar
M155T, E184K, E182K	–	Familial HCM	*Likely pathogenic*	
R146S	–	Cardiomyopathy	*Likely pathogenic*	
R162L	–	RCM	*Likelypathogenic*	
K174N	–		*Pathogenic*	

HCM: Hypertrophic Cardiomyopathy; RCM: Restrictive Cardiomyopathy; DCM: Dilated Cardiomyopathy.

**Table 5:** Cardiac troponin T (cTnT) pathogenic/likely pathogenic mutations.

Protein change	Molecular effects	Condition(s)	ACMG classifications	References
D270N	D270N-cTnT mutation impaired cTnC-cTnI and cTnC-cTnT interactions and decreased calcium sensitivity of myofilaments, decreased maximal ATPase activity and myofilament sliding speed, decreased cooperativity	LVNC	*Pathogenic*	149
ΔK210	All mutants modified the Ca^2+^ sensitivity of thin filament	DCM	*Pathogenic/Likely pathogenic*	106, 110, 149,109
R205L	R205L-cTnT mutant impaired cTnI-cTnC and cTnC-cTnT interactions and decreased calcium sensitivity of myofilaments decreased maximal ATPase activity, and myofilament sliding speed	Familial DCM	*Pathogenic*	149, 150
R173Q	The mutation R173Q decreased the rate of degradation of cTnT by calpain	Primary DCM	*Pathogenic/Likely pathogenic*	151
R173W	TnT-R173W mutation decreased PKA-mediated TnI phosphorylation at Ser-23/24 by limiting the binding of PKA to sarcomere.	DCM	*Pathogenic*	103, 149
E163K	The E163K-TnT mutation produced an elevated activation of the ATPase activity in reconstituted thin filaments.	Familial HCM	*Pathogenic/Likely pathogenic*	27
R141W	R141W-cTnT mutation increased the affinity of cTnT for alpha-tropomyosin and decreased calcium sensitivity of myofilaments, decreased maximal ATPase activity, and myofilament sliding speed, dissociation between calcium sensitivity, and PKA-mediated β-adrenergic response to cTnI phosphorylation	DCM	*Pathogenic*	149, 152
R151G, R159G, R205W	These mutations decreased calcium sensitivity of force development.	Primary DCM	*Pathogenic/Likely pathogenic*	148
R134G	R134G-cTnT mutant modified Ca^2+^ sensitivity of thin filament.	DCM	*Pathogenic/Likely pathogenic*	103, 149
R139H	R139H-cTnT mutant decreased Ca^2+^ sensitivity and maximal force development.	DCM	*Pathogenic/Likely pathogenic*	149, 153
R131W	Decreased Ca^2+^ sensitivity, decreased maximal ATPase activity, and myofilament sliding speed.	Primary DCM	*Pathogenic/Likely pathogenic*	149
R126W, R101W	Decreased Ca^2+^ sensitivity, decreased maximal ATPase activity, and myofilament sliding speed.	LVNC, Familial RCM, Familial HCM, Primary DCM	*Pathogenic/Likely pathogenic*	149
F110L	F110L-cTnT mutation interferes with the cTnT-tropomyosin biding.	HCM	*Pathogenic/Likely pathogenic*	154
F110I	F110I-TnT mutation increased Ca^2+^ sensitivity of force and ATPase activity.	HCM	*Pathogenic*	155
ΔE96, ΔE106	ΔE96-cTnT and ΔE106-cTnT fibers showed a definite inability to inhibit ATPase activity and a profound inability to decrease unregulated force after TnT displacement and TnI·TnC reconstitution.	RCM	*Pathogenic*	55
E96K	–	LVNC	*Pathogenic*	107
R94C	R94C-cTnT mutant demonstrated impaired cardiac regulation at the molecular level attributed to reduced Ca^2+^-dependent blocking of myosin's interaction with the thin filament	Familial RCM	*Pathogenic/Likely pathogenic*	157,156
K273E	K273E-cTnT mutant decreased ATPase activity and Ca^2+^ sensitivity and impaired force production.	DCM	*Pathogenic*	20
R92Q, E163R	D96A-cTnT mutation deregulated the interaction between cTnT and tropomyosin.	HCM	*Pathogenic/Likely pathogenic*	154
I79N	The mutation was involved in the alteration of the TPM binding domain of cTnT	Familial HCM, Familial RCM, LVNC	*Pathogenic*	27
W287∗, W294∗, W281∗, W284∗, W254∗, W297∗	–	Familial RCM, LVNC, Familial HCM	*Pathogenic/Likely pathogenic*	https://www.ncbi.nlm.nih.gov/clinvar
Q282fs, Q266fs, Q269fs, Q272fs, Q279fs, Q239fs	–	Cardiomyopathy	*Pathogenic*	
D277N, D267N, D280N, D237N, D264N, R212L, R202L, R172L, R199L, R215L, R143L, R168L, R173L, R183L, E106K, E91K	–	LVNC	*Pathogenic*	
N269K, N276K, N236K, N263K, N266K, N279K, ΔE91, K103Q, K93Q, K88Q	–	HCM	*Likely pathogenic*	
R196W, R203W, R206W, R193W, R190W, R163W	–	Familial HCM, LVNC, Familial RCM	*Pathogenic*	
S179F, S189F, S149F, S174F	–	HCM, LVNC, Familial HCM, Familial RCM,	*Pathogenic/Likely pathogenic*	
R183Q, R143Q, R168Q	–	LVNC, Familial HCM, Familial RCM, Primary DCM	*Pathogenic/Likely pathogenic*	
ΔE163, ΔE173, ΔE133, ΔE158	–	LVNC, HCM, Familial RCM, Familial HCM,	*Pathogenic/Likely pathogenic*	
E173K, E133K, E158K	–	LVNC, Familial HCM, Familial RCM	*Likely pathogenic*	
R151W, R111W, R136W	–	LVNC, HCM, Familial RCM, Familial HCM, Familial isolated DCM, Primary DCM	*Pathogenic*	
R131Q, R141Q, R101Q, R126Q	–	Familial HCM, Familial RCM, Primary DCM, LVNC	*Pathogenic*	
R130C, R140C, R125C, R100C	–	Familial HCM, Familial RCM, LVNC, HCM	*Pathogenic*	
E114Q, E119Q, E129Q, E118K, E128K, E113K, H104Y, H109Y, H119Y, D97E, D83E	–	Primary DCM	*Likely pathogenic*	
F120L, F105L	–	HCM, Wolff-Parkinson-White syndrome, Primary familial HCM	*Pathogenic/Likely pathogenic*	
F120I, F105I	–	LVNC, Familial HCM	*Pathogenic*	
R104L, R94L, R89L R104H, R94H, R89H	–	LVNC, Familial HCM, Familial RCM	*Pathogenic/Likely pathogenic*	
R104C, R89C	–	LVNC, Familial RCM, Familial HCM, Primary familial HCM	*Pathogenic/Likely pathogenic*	
R102Q, R87Q	–	HCM, LVNC, Familial HCM, Familial RCM, Familial HCM	*Pathogenic/Likely pathogenic*	
R102W, R92W, R87W	–	HCM, LVNC, Familial HCM, Primary familial HCM, Familial RCM	*Pathogenic*	
D86A, D81A, D95A	–	HCM, LVNC Familial HCM, Familial RCM	*Pathogenic/Likely pathogenic*	

LVNC: Left Ventricular Non-Compaction Cardiomyopathy; DCM: Dilated Cardiomyopathy; RCM: Restrictive Cardiomyopathy; HCM: Hypertrophic Cardiomyopathy.

At least 115 variants of TNNC1 due to point mutations that vary from wildtype protein (161 aa) by a single amino acid are reported. Approximately, 26% of these variants can compromise the Ca^2+^ sensitivity of cTnC or the ability of troponin to interact properly with the other cTn components. However, the possible adverse effects of all variants are unknown.^40^ The identified cTnC variants, the associated cardiomyopathies, and the relative molecular effects are reported in Table 3. Among them, the cTnC-A8V variant was associated with HCM and RCM.^40^^,^^90^ In *in vitro* studies, a delay of mechanical relaxation and intracellular Ca^2+^ decay was observed in cardiomyocytes with cTnC-A8V substitution.^90^ This is probably due to the replacement of alanine with valine in position 8 which may increase the Ca^2+^-binding affinity of cTnC, thus inducing a delay in the Ca^2+^ dissociation rate from the thin filament.^40^^,^^90^ Similarly, in an animal model of HCM containing the human cTnC-A8V substitution, an increase in Ca^2+^ sensitivity of cTnC was sufficient to induce cardiac remodeling and myofilament dysfunction.^91^ cTnC-A8V mice showed a thicker left ventricular wall and a lower diastolic diameter and volume compared with the control.^92^ Moreover, the *in vivo* study suggested that these adverse morphological changes affected more females than males.^92^ Alternatively, a reduction of Ca^2+^ sensitivity could be the primary consequence of those cTnC variants that are associated with DCM, such as D75Y, E59D, and E32K.^93^^,^^94^ In particular, the D75Y substitution, but not E59D, causes a reduction of the Ca^2+^ affinity. However, both D75Y and E59D are required to reduce the actomyosin ATPase activity and maximal force development in muscle fibers, indicating that E59D substitution can be considered as a booster for D75Y effects.^94^

TNNI3 mutations account for about 5% of genotyped families with HCM.^94^, ^95^, ^96^, ^97^, ^98^ A list of TNNI3 mutations together with the associated molecular and pathophysiological effects (when known) is reported in Table 4. The main mutations of the cTnI gene are located in the inhibitory/switch peptide and the C-terminal domain, thus interfering with the regulatory property of cTnI. It has been observed that the mutations R145G, R145Q, R162W, R21C, K206Q, and ΔK178 have the ability to interfere with Ca^2+^ sensitivity and force development by sarcomere fibers.^53^ The cTnI-R145G variant, located within the inhibitory peptide of cTnI, is one of the most studied TNNI3 mutations. The cTnI-R145G variant is historically associated with HCM, however, its effect on the cTn function is not fully clear.^36^ Enhanced Ca^2+^ sensitivity and actomyosin ATPase activities were observed in thin filaments reconstituted with human skeletal muscle, actin, tropomyosin, and recombinant human cTn, including cTnI with mutation R145G when compared with filaments with cTnI wild-type.^95^ However, when human recombinant cTnI-R145G was incorporated into murine or guinea-pig myofibrils, Ca^2+^sensitivity did not change,^95^, ^96^, ^97^, ^98^ whilst if mouse recombinant cTnI-R145G was merged in the murine sarcomere, a decrease in Ca^2+^ sensitivity could be observed.^95^ The cTnI-R21C substitution, also extensively studied,^99^ is located at the cTnI N-terminal region, near Ser 23 and Ser 24.^46^ The contractile fibers containing the TnI-R21C substitution are characterized by an increase in Ca^2+^ sensitivity and force development, and by a reduction in phosphorylation of Ser 23/24 by PKA when compared with wild-type fibers.^100^^,^^101^ Accordingly, in cTnI-R21C transgenic mice, a reduction in Ser 23/24 phosphorylation, an alteration in the β-adrenergic pathway, and an activation of hypertrophic molecular mechanisms were observed compared with control mice.^100^^,^^101^ Moreover, abnormalities in calcium regulation were observed using CalTrack, a MatLab-based algorithm able to monitor fluorescent calcium changes in living cardiomyocytes. In particular the cTnI-R21C^+/−^ variant caused a significant acceleration of time to reach calcium peak and a longer duration of time for Ca^2+^ transient decay and Ca^2+^ transient duration. Such abnormalities were only partially corrected by dedicated allosteric modulators of thick filament drugs, such as mavacamten.^102^

Mutations of cTns affecting the contact site can disrupt the normal troponin complex interaction, leading to a decreased affinity of binding between cTnI and cTnT.^103^^,^^104^ cTnI-C11R is a dominant negative mutation responsible for a decreased binding affinity of cTnI for TnT, thus reducing the regulatory function of the entire complex on the sarcomere.^104^

The known pathogenic/likely pathogenic mutations of TNNT2, including deletions (protein change in ΔE163, ΔE173, ΔK210, ΔK177, *etc*.) and single nucleotide substitutions (protein change in R134G, R134S, R92L, R92W, R94C, K103Q, K88Q, I79N, I89N, *etc*.), are reported in Table 5. Most of the cTnT mutations induced changes either in the flexibility of cTnT or in the Ca^2+^ affinity for the cTnC calcium-binding site, thus contributing to HCM, RCM, DCM, or left ventricular non-compaction cardiomyopathy phenotypes.^105^, ^106^, ^107^, ^108^, ^109^, ^110^ The location of the cTnT mutation contributes to the type of alterations in protein properties.^108^^,^^109^ Mutations in the T2 domain, such as the cTnT-ΔK210 mutation, historically associated with dilated ventricular chamber and reduced systolic function, can directly contribute to impairment in Ca^2+^ affinity of cTnC.^98^ Ca^2+^ sensitivity of the recombinant thin filaments containing an equimolar mix of cTnT-ΔK210 and cTnT-wild-type decreased compared with control filaments.^71^ Moreover, the thin filament sliding speed was reduced in recombinant thin filaments containing cTnT-ΔK210 compared with wild-type cTnT.^108^ Considering that most of the TNNT2 mutations are heterozygous, these data could suggest that cTnT-ΔK210 mutation contributes in a dose-dependent fashion to DCM development.^108^ In a recent study using human embryonic stem cell-derived cardiomyocytes and mouse heart tissues, it was observed that cTnT-ΔK210 mutation was associated with a reduced expression of Xin actin-binding repeat-containing protein genes.^111^ The XIN proteins are localized predominantly at the intercalated discs and are responsible for signal transduction involved in cardiac remodeling.^111^ A reduction of XIN proteins is associated with impaired contractility and attenuated muscle repair.^111^ *In vivo* and *in vitro* studies showed that the overexpression of XIN proteins improved sarcomere organization and contraction force of TNNT2-ΔK210, and significantly improved the DCM phenotypes, suggesting a possible therapeutic target for subjects carrying the cTnT-ΔK210 mutation.^111^

Mutations in the T1 domain could be associated with an aberrant interaction with tropomyosin, as observed for cTnT-R94C, leading to a reduced binding affinity of cTnT for thin filaments.^112^ The mutation cTnT-R134G, located in the T1 domain of cTnT, is typically associated with DCM. In an *in vitro* study, cardiomyocytes with cTnT-R134G substitution showed a reduction in Ca^2+^ sensitivity and contractile function together with sarcomere disorganization when compared with the control.^112^ However, the regular thin filament with cTnT-R134G substitution showed an increased cTnC Ca^2+^ affinity when compared with the wild-type.^112^ Discrepancies in these results could depend on the ability of cTnT-R134G to promote shifting of the Tm in a permanent locking state independently from Ca^2+^ concentration, thus allowing a reduction of the contractile function of the sarcomere.^112^ Again, cTnT-R173W substitution is typically associated with DCM. This mutation modifies the interaction between the N-terminal domain of cTnT and Tm, thus reducing the troponin anchoring on sarcomere filaments and destabilizing the sarcomere protein alignment. Moreover, TnT-R173W substitution decreases PKA-mediated TnI phosphorylation at Ser 23/24 by limiting the binding of PKA to sarcomere. PKA-mediated phosphorylation is a critical regulatory step for the modulation of cardiac contraction.^108^

### Regulation of gene expression

During HF, fetal isoforms of cTnT or TnI are expressed together with the adult isoforms. The co-presence of different cTnT and TnI isoforms is associated with a reduction of cardiac performance, a decrease in stroke volume, and slower velocities of contraction and relaxation, thus inducing a progression of HF.^37^^,^^79^^,^^80^ A recent study reported the re-expression of fetal isoforms of cTnT and TnI in cardiac biopsies collected from adult and children patients with HF before ventricular assist device implant.^80^ However, fetal isoforms were not modified by ventricular assist device implant, while an increased expression of adult isoforms, such as cTnT3, cTnT4, and cTnI, was observed in pediatric patients.^80^

The effects of different isoforms of cTnT (1/3/4) in combination with ssTnI or cTnI on Ca^2+^ sensitivity and force of contraction of sarcomere were studied.^79^ In the presence of ssTnI, the absence of exon 5 in cTnT (cTnT3 or cTnT4) decreased the Ca^2+^ sensitivity compared with cTn with cTnT1. Interestingly, fibers with cTnT4-ssTnI and cTnT4-cTnI developed similar maximal force. In contrast, the maximal force of contraction was higher in cTnT3-ssTnI fiber than in cTnT3-cTnI recombinant skinned fiber.^63^ It is unlikely that the increase of maximal force was due to ssTnI alone, but rather it was dependent on some complex interplay between the cTnT isoforms, TnI isoforms, and the rest of the thin filament proteins.^79^

The ability of cTnI to regulate the actomyosin ATPase activity was similar in the presence either of cTnT3 or of cTnT4, while ssTnI linked with cTnT3 showed an increased ATPase activity when compared with the complex ssTnI-cTnT4.^79^

cTnC plays a critical role in regulating muscle contraction by its ability to bind Ca^2+^ to the EF-hand II regulatory site.^113^ The effects of cTnT or TnI isoforms on the rate of Ca^2+^ removal from the high- or low-affinity sites on cTnC were investigated. cTnT1 had a greater effect on the kinetics of the Ca^2+^ dissociation rate from site II of cTnC in the cTnI-cTnC complex than in the ssTnI-cTnC complex. In particular, the cTn containing ssTnI had a 2-to-3-fold slower rate of Ca^2+^ dissociation from cTnC (site II) than the complex containing cTnI.^79^ These results suggest that in the normal fetal heart, a contemporary presence of cTnT1 isoform, which showed a high Ca^2+^ sensitivity, and ssTnI, which had a slower rate of Ca^2+^ dissociation, increased the duration of the systolic phase. These characteristics of fetal cTn (cTnT1-ssTnI-cTnC) could be associated with the lower Ca^2+^ levels in the fetal heart. Thus, the re-expression of the fetal gene of troponin in HF patients and their substitution to functional cTn complex could be associated with the disarray of sarcomere and reduction of left ventricular ejection fraction typically observed in these patients.^37^^,^^80^^,^^99^ The causal relationship between the re-expression of fetal cTn genes and sarcomere disarray is one of the possibilities to be explored to better understand the molecular mechanisms underlying HF.

Moreover, TNNT2 is expressed in cardiomyocytes during heart and skeletal muscle development and it can be re-expressed in the muscle during neuromuscular diseases.^114^^,^^115^ In these patients, in the absence of myocardial injury, a persistent blood elevation of cTnT without cTnI was observed. Re-expressed cTnT in diseased skeletal muscle is considered the source of the elevated cTnT detected in the circulation of these patients.^114^

Very recently, an epigenetic regulation of cTn genes in pediatric patients with HF supported by a ventricular assist device was reported. Specifically, gene expression of troponins, found to be differentially expressed in cardiac biopsies at post-ventricular assist device when compared with pre-ventricular assist device, were identified as putative targets of miR-199b-5p, miR-19a-3p, and miR-1246.^80^

Moreover, regulation of troponin expression during translation has been reported. A very recent study in RPL3L-deficient mice suggested that loss of RPL3L ribosomes determined a reduction of cardiac contractility as a consequence of loss of the overall transcriptome. In particular, transcripts from genes related to sarcomere including cTnT-mRNA were affected by RPL3L mutation,^3^^,^^116^ thus facilitating the heart remodeling and dysfunction. During HF an increase in 2-oxoglutarate- and iron-dependent oxygenase domain-containing protein 1 (OGFOD1), a member of the 2-oxoglutarate-dependent dioxygenase family, that regulates several aspects of gene expression including translation, was found to be up-regulated. *In vitro* and *in vivo* studies reported that loss of OGFOD1 (in iPCS-CMs OGFOD1-KO or OGFOD1-KO mice) resulted in a significantly increased level of sarcomere proteins, such as titin, cTnC, cTnI, and cTnT, compared with control.^117^^,^^118^ Moreover, OGFOD1-KO mice were protected from hypertrophy under chronic stress conditions.^117^

### Post-translational modifications

As previously described, the phosphorylation of Ser 23/24 can be considered among the main PTM associated with cTnI, possibly leading to functional alteration. During HF, the pathway catecholamine/β-adrenergic receptor/PKA decreased as a consequence of β-adrenergic receptor down-regulation. In particular, a reduction of both β-adrenergic receptors I and II was observed in cardiac biopsies obtained from end-stage DCM patients compared with control. Thus, in DCM patients, the turn-off of the β-adrenergic receptors was directly related to the reduction of PKA activity and Ser 23/24 phosphorylation.^119^ Consequently, this reduction induced a slow sarcomere relaxation and an impaired heart function.^111^ Conversely, the expression of the N-terminal-deleted cTnI (cTnI-ND) decreased the development of the cardiomyopathy-like phenotype in a β-adrenergic-deficient transgenic aged mouse model.^120^^,^^121^ Thus, the removal of cTnI N-terminal extension induces structural and functional modifications in cTnI molecules such as those typically observed in case of low Ser 23/24 phosphorylation. These data suggested that the removal of the N-terminal region may maintain cardiac function in aged mice during stress.^120^^,^^121^

Besides reduction in PKA activity, the hyperactivation of Ca^2+^/calmodulin-dependent protein kinase II during long-lasting hyperglycemia causes phosphorylation of cTnI and decreases myofilament Ca^2+^ sensitivity.^59^

To evaluate the involvement of cTnT phosphorylation in pathological conditions, such as HF, the PTMs of cTnT and other sarcomere proteins were compared between end-stage HF patients and non-failing donor hearts.^84^^,^^19^ cTnI and myosin light chain 2 phosphorylation decreased in failing human myocardium, while cTnT phosphorylation was unaltered.^122^ The Ca^2+^ sensitivity and the myofilament isometric force increased in HF patients compared with the control.^123^^,^^124^

In rats with congestive HF, phosphorylation of cTnT and cTnI was greater in the failing heart compared with the control and higher levels of phosphorylated cTnT were present in the failing left ventricle compared with the right ventricle. In addition, the phosphorylation of cTnT is associated with a reduction of Ca^2+^ sensitivity and, consequently, with dysfunction of myofilament. Overall, these studies showed a discrepancy between human and animal models, emphasizing the need for other specific studies dedicated to humans.

### Small molecules: A new era of cardiomyopathy therapy

The development of drugs targeting the contractile apparatus of cardiac muscle is considered the new frontier for DCM, HCM, and, consequently, HF treatment. Five classes of “small molecules” have been identified and classified based on their effects. Myosin inhibitors, Ca^2+^-desensitizers, and recoupler were designed to treat hypercontractility,^125^, ^126^, ^127^ and myosin activators and Ca^2+^-sensitizers were proposed as positive inotrope agents.^128^^,^^129^

Among the agents for treating hypercontractility, mavacamten is the first and only reversible cardiac myosin inhibitor approved by the FDA.^130^ This small molecule can influence cardiac muscle contractility, reducing dynamic left ventricular outflow tract obstruction and improving cardiac filling pressures in patients with HCM. cTn is suggested as a target of Ca^2+^-desensitizer small molecules in hypercontractility conditions. Among them, *in vitro* studies suggested a cardioprotective role for epigallocatechin-3-gallate and epicatechin gallate^131^, ^132^, ^133^ which were able to improve the diastolic function in isolated working mouse hearts, binding cTnC and reducing Ca^2+^ sensitivity of troponin.^132^ However, these molecules showed also pleiotropic effects that make them inadequate for *in vivo* use. Another class of small molecules able to interfere with cTn function are recouplers. These new small molecules demonstrated the ability to restore the phosphorylation-dependent Ca^2+^-sensitivity in HCM and DCM conditions dependent on cTnI mutations.^17^

The hypo-contractility of the heart is a complex condition associated with HF. Considering the complex nature of hypo-contractility appears evident, several elements of the contractile apparatus should be at the same time targeted by new treatments to have an efficacy clinical response. Currently, a fair number of myosin activators and Ca^2+^-sensitizers were proposed as positive inotrope agents.^128^^,^^129^ Omecamtiv mecarbil is a first class of myosin activators, a small molecule designed to increase the number of active actin-myosin cross bridges during the cardiac cycle. Even if an improvement in cardiac contraction was observed in preclinical studies, to date no clinical trials have obtained FDA approval.^112^^,^^134^ Among Ca^2+^-sensitizers, bepridil and EMD57033 in a different way, were able to improve the force contractility of sarcomere modulating the interaction between the N-terminal region of cTnI and C-terminal domain of cTnC, independently from Ser 23/24 PTMs on cTnI.^135^ The AGM 594 novel small-molecule that acts as a troponin activator can increase contractility *ex vivo* and *in vivo* in anesthetized normal rats without negative effects on myocardial energetics.^129^ Among them, only AMG 594 demonstrated more positive effects than adverse effects in preclinical trials suggesting that more investigation is needed before replacing the current inotrope therapy with these troponin activators.

## Conclusion

Because cardiac troponins are regulatory elements of sarcomere function, any conditions that may affect their synergic interaction could interfere with a proper myofilament function, resulting in heart dysfunction, cardiomyopathy, or death. Further investigations are needed to complete the knowledge of the regulatory complex of sarcomere. It is still questioned how a modification within the thin filament could modulate the contractile function and whether a specific change in cTn is directly responsible or is indirectly an effector of additional downstream events for adverse remodeling and dysfunction.

To date, several mutations of TNNC1, TNNI3, and TNNT2 genes have been identified, but the physio-pathological effect of each mutation has not yet been clarified. Even if sarcomere variants generally have high penetrance, they produce a variable expression of clinical manifestations.^71^ Mutations in the cTnI gene have been identified in families with HCM, RCM, and recessively inherited idiopathic dilated cardiomyopathy^136^, ^137^, ^138^, ^139^ reflecting the important and diverse functional role of the cTnI protein in normal cardiac biology. The diversity of the phenotypic expression of cTns mutations is not explained by the current knowledge of their molecular and functional impact and suggests that additional environmental, genetic, and epigenetic factors may interact and affect clinical disease expression leading to phenotypic diversity of cardiomyopathies. A better knowledge of molecular mechanisms affected by these mutations has important implications for understanding disease pathogenesis and could be useful for the development of targeted therapeutics for those patient subpopulations with myofilament defects.

Accordingly, the switch of cTnI and cTnT from fetal to adult isoforms during physiological development or the re-expression of fetal troponin isoforms during heart diseases, such as HF, has been documented and a possible regulatory role for epigenetic mechanisms (*i.e*., miRNA) has been proposed. However, only a few studies investigated the link between epigenetic mechanisms and cardiac troponin gene expression. A complete understanding of how and which miRNAs are involved in cardiac troponin expression during physiological and pathological conditions is crucial for hypothesizing the use of miRNA therapy in HF patients.

Moreover, X-ray crystallography and CryoEM technology have improved the knowledge of the structure and spatial orientation of every single domain of cTn, but gaps in understanding the cTn switching mechanism and movement across the actin during cardiac muscle activation are still present.

In addition to the well-known role of cTns as regulatory components of the sarcomere, recent studies have demonstrated the presence of cTnI and cTnT in the nucleus of cardiomyocytes. Actually, the mechanisms that regulate the transport of cTnI and cTnT from the cytoplasm into the nucleus are not known, while an interaction of cTnI with a histone deacetylase^140^ and of cTnT with histone de-methylases was observed in the nucleus of cardiomyocytes.^141^ Thus, these results indicate a novel role of cTns and might provide new aspects for investigations in heart development and cardiovascular diseases.

Furthermore, many additional questions remain unsolved: how many PTMs ascertained *in vitro* could be detected *in vivo*? What are their functions? What is the role of non-canonical cTnT? Answers to these questions could help to identify new drugs targeting the pathways involved in post-translational cTn modifications, opening a new era for cardiomyopathy therapy, with the final aim of improving heart function.

## Conflict of interests

The authors declare there are no competing interests.

## References

[1] Spudich J.A. (2001). The myosin swinging cross-bridge model. Nat Rev Mol Cell Biol.

[2] Fabiato A., Fabiato F. (1978). Effects of pH on the myofilaments and the sarcoplasmic reticulum of skinned cells from cardiac and skeletal muscles. J Physiol.

[3] Thomson E., Ferreira-Cerca S., Hurt E. (2013). Eukaryotic ribosome biogenesis at a glance. J Cell Sci.

[4] Ebashi S. (1961). Calcium binding activity of vesicular relaxing factor. J Chir.

[5] Papadaki M., Marston S.B. (2016). The importance of intrinsically disordered segments of cardiac troponin in modulating function by phosphorylation and disease-causing mutations. Front Physiol.

[6] Ringer S. (1883). A further contribution regarding the influence of the different constituents of the blood on the contraction of the heart. J Physiol.

[7] Ramirez-Correa G.A., Jin W., Wang Z. (2008). O-linked GlcNAc modification of cardiac myofilament proteins: a novel regulator of myocardial contractile function. Circ Res.

[8] Kuo I.Y., Ehrlich B.E. (2015). Signaling in muscle contraction. Cold Spring Harbor Perspect Biol.

[9] Rall J.A. (2019). Calcium and muscle contraction: the triumph and tragedy of Lewis Victor Heilbrunn. Adv Physiol Educ.

[10] Cheng Y., Lindert S., Kekenes-Huskey P. (2014). Computational studies of the effect of the S23D/S24D troponin I mutation on cardiac troponin structural dynamics. Biophys J.

[11] Parvatiyar M.S., Landstrom A.P., Figueiredo-Freitas C., Potter J.D., Ackerman M.J., Pinto J.R. (2012). A mutation in TNNC1-encoded cardiac troponin C, TNNC1-A31S, predisposes to hypertrophic cardiomyopathy and ventricular fibrillation. J Biol Chem.

[12] Streng A.S., de Boer D., van der Velden J., van Dieijen-Visser M.P., Wodzig W.K.W.H. (2013). Posttranslational modifications of cardiac troponin T: an overview. J Mol Cell Cardiol.

[13] Zamora J.E., Papadaki M., Messer A.E., Marston S.B., Gould I.R. (2016). Troponin structure: its modulation by Ca^2+^ and phosphorylation studied by molecular dynamics simulations. Phys Chem Chem Phys.

[14] Bremel R.D., Weber A. (1972). Cooperation within actin filament in vertebrate skeletal muscle. Nat New Biol.

[15] Annemarie Weber Franzini-Armstrong C. (1998). Ca^2+^ and the regulation of muscle contraction. Trends Cell Biol.

[16] Murray J.M., Weber A. (1974). The cooperative action of muscle proteins. Sci Am.

[17] Weber A., Murray J.M. (1973). Molecular control mechanisms in muscle contraction. Physiol Rev.

[18] Geeves M.A., Lehrer S.S. (1994). Dynamics of the muscle thin filament regulatory switch: the size of the cooperative unit. Biophys J.

[19] Martin-Garrido A., Biesiadecki B.J., Salhi H.E. (2018). Monophosphorylation of cardiac troponin-I at *Ser*-23/24 is sufficient to regulate cardiac myofibrillar Ca^2+^ sensitivity and calpain-induced proteolysis. J Biol Chem.

[20] Fujino N., Shimizu M., Ino H. (2002). A novel mutation Lys273Glu in the cardiac troponin T gene shows high degree of penetrance and transition from hypertrophic to dilated cardiomyopathy. Am J Cardiol.

[21] Lehrer S.S., Geeves M.A. (1998). The muscle thin filament as a classical cooperative/allosteric regulatory system. J Mol Biol.

[22] Mijailovich S.M., Li X., Griffiths R.H., Geeves M.A. (2012). The Hill model for binding myosin S1 to regulated actin is not equivalent to the McKillop-Geeves model. J Mol Biol.

[23] Moss R.L., Razumova M., Fitzsimons D.P. (2004). Myosin crossbridge activation of cardiac thin filaments: implications for myocardial function in health and disease. Circ Res.

[24] Risi C.M., Pepper I., Belknap B. (2021). The structure of the native cardiac thin filament at systolic Ca^2+^ levels. Proc Natl Acad Sci U S A.

[25] Tobacman L.S. (2021). Troponin revealed: Uncovering the structure of the thin filament on-off switch in striated muscle. Biophys J.

[26] Yamada Y., Namba K., Fujii T. (2020). Cardiac muscle thin filament structures reveal calcium regulatory mechanism. Nat Commun.

[27] Szczesna D., Zhang R., Zhao J., Jones M., Guzman G., Potter J.D. (2000). Altered regulation of cardiac muscle contraction by troponin T mutations that cause familial hypertrophic cardiomyopathy. J Biol Chem.

[28] Wang Z., Raunser S. (2023). Structural biochemistry of muscle contraction. Annu Rev Biochem.

[29] Pavadai E., Rynkiewicz M.J., Ghosh A., Lehman W. (2020). Docking troponin T onto the tropomyosin overlapping domain of thin filaments. Biophys J.

[30] Herrmann J., Haude M., Lerman A. (2001). Abnormal coronary flow velocity reserve after coronary intervention is associated with cardiac marker elevation. Circulation.

[31] Kleinbongard P., Heusch G. (2022). A fresh look at coronary microembolization. Nat Rev Cardiol.

[32] Quast C., Kober F., Becker K. (2022). Multiparametric MRI identifies subtle adaptations for demarcation of disease transition in murine aortic valve stenosis. Basic Res Cardiol.

[33] Liu X., Li M., Chen Z. (2022). Mitochondrial calpain-1 activates NLRP3 inflammasome by cleaving ATP5A1 and inducing mitochondrial ROS in CVB_3_-induced myocarditis. Basic Res Cardiol.

[34] Thielmann M., Kottenberg E., Kleinbongard P. (2013). Cardioprotective and prognostic effects of remote ischaemic preconditioning in patients undergoing coronary artery bypass surgery: a single-centre randomised, double-blind, controlled trial. Lancet.

[35] Ho C.Y., Charron P., Richard P., Girolami F., Van Spaendonck-Zwarts K.Y., Pinto Y. (2015). Genetic advances in sarcomeric cardiomyopathies: state of the art. Cardiovasc Res.

[36] Kimura A. (2016). Molecular genetics and pathogenesis of cardiomyopathy. J Hum Genet.

[37] Anderson P.A., Malouf N.N., Oakeley A.E., Pagani E.D., Allen P.D. (1991). Troponin T isoform expression in humans. A comparison among normal and failing adult heart, fetal heart, and adult and fetal skeletal muscle. Circ Res.

[38] Hinken A.C., Solaro R.J. (2007). A dominant role of cardiac molecular motors in the intrinsic regulation of ventricular ejection and relaxation. Physiology.

[39] Katrukha I.A. (2013). Human cardiac troponin complex. Structure and functions. Biochemistry.

[40] Reinoso T.R., Landim-Vieira M., Shi Y. (2021). A comprehensive guide to genetic variants and post-translational modifications of cardiac troponin C. J Muscle Res Cell Motil.

[41] Marston S., Zamora J.E. (2020). Troponin structure and function: a view of recent progress. J Muscle Res Cell Motil.

[42] Hunkeler N.M., Kullman J., Murphy A.M. (1991). Troponin I isoform expression in human heart. Circ Res.

[43] Saggin L., Gorza L., Ausoni S., Schiaffino S. (1989). Troponin I switching in the developing heart. J Biol Chem.

[44] Sasse S., Brand N.J., Kyprianou P. (1993). Troponin I gene expression during human cardiac development and in end-stage heart failure. Circ Res.

[45] Huang X., Pi Y., Lee K.J. (1999). Cardiac troponin I gene knockout: a mouse model of myocardial troponin I deficiency. Circ Res.

[46] McConnell B.K., Moravec C.S., Bond M. (1998). Troponin I phosphorylation and myofilament calcium sensitivity during decompensated cardiac hypertrophy. Am J Physiol.

[47] Yang B., Zhao H., Dong R. (2020). MiR-449 improves cardiac function by regulating HDAC1 and cTnI. Eur Rev Med Pharmacol Sci.

[48] Pan B., Xu Z.W., Xu Y. (2016). Diastolic dysfunction and cardiac troponin I decrease in aging hearts. Arch Biochem Biophys.

[49] Pan B., Quan J., Liu L. (2017). Epigallocatechin gallate reverses cTnI-low expression-induced age-related heart diastolic dysfunction through histone acetylation modification. J Cell Mol Med.

[50] van Rooij E., Quiat D., Johnson B.A. (2009). A family of microRNAs encoded by myosin genes governs myosin expression and muscle performance. Dev Cell.

[51] van Rooij E., Sutherland L.B., Qi X., Richardson J.A., Hill J., Olson E.N. (2007). Control of stress-dependent cardiac growth and gene expression by a microRNA. Science.

[52] Villar-Palasi C., Kumon A. (1981). Purification and properties of dog cardiac troponin T kinase. J Biol Chem.

[53] Cimiotti D., Fujita-Becker S., Möhner D. (2020). Infantile restrictive cardiomyopathy: cTnI-R170G/W impair the interplay of sarcomeric proteins and the integrity of thin filaments. PLoS One.

[54] Gordon A.M., Homsher E., Regnier M. (2000). Regulation of contraction in striated muscle. Physiol Rev.

[55] Pinto J.R., Parvatiyar M.S., Jones M.A., Liang J., Potter J.D. (2008). A troponin T mutation that causes infantile restrictive cardiomyopathy increases Ca^2+^ sensitivity of force development and impairs the inhibitory properties of troponin. J Biol Chem.

[56] Lehman W., Pavadai E., Rynkiewicz M.J. (2021). C-terminal troponin-I residues trap tropomyosin in the muscle thin filament blocked-state. Biochem Biophys Res Commun.

[57] Pearlstone J.R., Smillie L.B. (1985). The interaction of rabbit skeletal muscle troponin-T fragments with troponin-I. Can J Biochem Cell Biol.

[58] Oda T., Yanagisawa H., Wakabayashi T. (2020). Cryo-EM structures of cardiac thin filaments reveal the 3D architecture of troponin. J Struct Biol.

[59] Miura M., Hasegawa T., Matsumoto A. (2021). Effect of transient elevation of glucose on contractile properties in non-diabetic rat cardiac muscle. Heart Ves.

[60] McKillop D.F., Geeves M.A. (1993). Regulation of the interaction between actin and myosin subfragment 1: evidence for three states of the thin filament. Biophys J.

[61] Kiani F.A., Lehman W., Fischer S., Rynkiewicz M.J. (2019). Spontaneous transitions of actin-bound tropomyosin toward blocked and closed states. J Gen Physiol.

[62] Lehman W. (2017). Switching muscles on and off in steps: the McKillop-geeves three-state model of muscle regulation. Biophys J.

[63] Malnic B., Farah C.S., Reinach F.C. (1998). Regulatory properties of the NH_2_- and COOH-terminal domains of troponin T. ATPase activation and binding to troponin I and troponin C. J Biol Chem.

[64] Solaro R.J., Kobayashi T. (2011). Protein phosphorylation and signal transduction in cardiac thin filaments. J Biol Chem.

[65] Biesiadecki B.J., Westfall M.V. (2019). Troponin I modulation of cardiac performance: plasticity in the survival switch. Arch Biochem Biophys.

[66] Yasuda S.I., Coutu P., Sadayappan S., Robbins J., Metzger J.M. (2007). Cardiac transgenic and gene transfer strategies converge to support an important role for troponin I in regulating relaxation in cardiac myocytes. Circ Res.

[67] Nixon B.R., Thawornkaiwong A., Jin J. (2012). AMP-activated protein kinase phosphorylates cardiac troponin I at *Ser*-150 to increase myofilament calcium sensitivity and blunt PKA-dependent function. J Biol Chem.

[68] Zhang R., Zhao J., Mandveno A., Potter J.D. (1995). Cardiac troponin I phosphorylation increases the rate of cardiac muscle relaxation. Circ Res.

[69] Dong W.J., Jayasundar J.J., An J., Xing J., Cheung H.C. (2007). Effects of PKA phosphorylation of cardiac troponin I and strong crossbridge on conformational transitions of the N-domain of cardiac troponin C in regulated thin filaments. Biochemistry.

[70] Takimoto E., Soergel D.G., Janssen P.M.L., Stull L.B., Kass D.A., Murphy A.M. (2004). Frequency- and afterload-dependent cardiac modulation *in vivo* by troponin I with constitutively active protein kinase A phosphorylation sites. Circ Res.

[71] Robertson S.P., Johnson J.D., Holroyde M.J., Kranias E.G., Potter J.D., Solaro R.J. (1982). The effect of troponin I phosphorylation on the Ca^2+^-binding properties of the Ca^2+^-regulatory site of bovine cardiac troponin. J Biol Chem.

[72] Baryshnikova O.K., Robertson I.M., Mercier P., Sykes B.D. (2008). The dilated cardiomyopathy G159D mutation in cardiac troponin C weakens the anchoring interaction with troponin I. Biochemistry.

[73] Burkart E.M., Sumandea M.P., Kobayashi T. (2003). Phosphorylation or glutamic acid substitution at protein kinase C sites on cardiac troponin I differentially depress myofilament tension and shortening velocity. J Biol Chem.

[74] Sakthivel S., Finley N.L., Rosevear P.R. (2005). *In vivo* and *in vitro* analysis of cardiac troponin I phosphorylation. J Biol Chem.

[75] Kirk J.A., MacGowan G.A., Evans C. (2009). Left ventricular and myocardial function in mice expressing constitutively pseudophosphorylated cardiac troponin I. Circ Res.

[76] Tobacman L.S. (1996). Thin filament-mediated regulation of cardiac contraction. Annu Rev Physiol.

[77] Manning E.P., Tardiff J.C., Schwartz S.D. (2012). Molecular effects of familial hypertrophic cardiomyopathy-related mutations in the TNT1 domain of cTnT. J Mol Biol.

[78] Gomes A.V., Guzman G., Zhao J., Potter J.D. (2002). Cardiac troponin T isoforms affect the Ca^2+^ sensitivity and inhibition of force development. Insights into the role of troponin T isoforms in the heart. J Biol Chem.

[79] Gomes A.V., Potter J.D. (2004). Cellular and molecular aspects of familial hypertrophic cardiomyopathy caused by mutations in the cardiac troponin I gene. Mol Cell Biochem.

[80] Ragusa R., Di Molfetta A., Del Turco S. (2021). Epigenetic regulation of cardiac troponin genes in pediatric patients with heart failure supported by ventricular assist device. Biomedicines.

[81] Biesiadecki B.J., Chong S.M., Nosek T.M., Jin J.P. (2007). Troponin T core structure and the regulatory NH_2_-terminal variable region. Biochemistry.

[82] Jaquet K., Fukunaga K., Miyamoto E., Meyer H.E. (1995). A site phosphorylated in bovine cardiac troponin T by cardiac CaM kinase II. Biochim Biophys Acta.

[83] Sumandea M.P., Vahebi S., Sumandea C.A., Garcia-Cazarin M.L., Staidle J., Homsher E. (2009). Impact of cardiac troponin T N-terminal deletion and phosphorylation on myofilament function. Biochemistry.

[84] He X., Liu Y., Sharma V. (2003). ASK1 associates with troponin T and induces troponin T phosphorylation and contractile dysfunction in cardiomyocytes. Am J Pathol.

[85] Vahebi S., Kobayashi T., Warren C.M., de Tombe P.P., Solaro R.J. (2005). Functional effects of rho-kinase-dependent phosphorylation of specific sites on cardiac troponin. Circ Res.

[86] Pfleiderer P., Sumandea M.P., Rybin V.O., Wang C., Steinberg S.F. (2009). Raf-1: a novel cardiac troponin T kinase. J Muscle Res Cell Motil.

[87] Sancho Solis R., Ge Y., Walker J.W. (2008). Single amino acid sequence polymorphisms in rat cardiac troponin revealed by top-down tandem mass spectrometry. J Muscle Res Cell Motil.

[88] Keyt L.K., Duran J.M., Bui Q.M. (2022). Thin filament cardiomyopathies: a review of genetics, disease mechanisms, and emerging therapeutics. Front Cardiovasc Med.

[89] Martin A.A., Thompson B.R., Hahn D. (2022). Cardiac sarcomere signaling in health and disease. Int J Mol Sci.

[90] Albury A.N.J., Swindle N., Swartz D.R., Tikunova S.B. (2012). Effect of hypertrophic cardiomyopathy-linked troponin C mutations on the response of reconstituted thin filaments to calcium upon troponin I phosphorylation. Biochemistry.

[91] Martins A.S., Parvatiyar M.S., Feng H.Z. (2015). *In vivo* analysis of troponin C knock-In (A8V) mice: evidence that TNNC1 is a hypertrophic cardiomyopathy susceptibility gene. Circ Cardiovasc Genet.

[92] Dieseldorff Jones K.M., Vied C., Valera I.C., Chase P.B., Parvatiyar M.S., Pinto J.R. (2020). Sexual dimorphism in cardiac transcriptome associated with a troponin C murine model of hypertrophic cardiomyopathy. Physiol Rep.

[93] Deng Y., Schmidtmann A., Redlich A., Westerdorf B., Jaquet K., Thieleczek R. (2001). Effects of phosphorylation and mutation R145G on human cardiac troponin I function. Biochemistry.

[94] Dweck D., Reynaldo D.P., Pinto J.R., Potter J.D. (2010). A dilated cardiomyopathy troponin C mutation lowers contractile force by reducing strong myosin-actin binding. J Biol Chem.

[95] Kruger M., Zittrich S., Redwood C. (2005). Effects of the mutation R145G in human cardiac troponin I on the kinetics of the contraction-relaxation cycle in isolated cardiac myofibrils. J Physiol.

[96] Sorrentino U., Gabbiato I., Canciani C. (2023). Homozygous *TNNI*_*3*_ mutations and severe early onset dilated cardiomyopathy: patient report and review of the literature. Genes.

[97] Cheng Y., Regnier M. (2016). Cardiac troponin structure-function and the influence of hypertrophic cardiomyopathy associated mutations on modulation of contractility. Arch Biochem Biophys.

[98] Burton D., Abdulrazzak H., Knott A. (2002). Two mutations in troponin I that cause hypertrophic cardiomyopathy have contrasting effects on cardiac muscle contractility. Biochem J.

[99] Gomes A.V., Harada K., Potter J.D. (2005). A mutation in the N-terminus of troponin I that is associated with hypertrophic cardiomyopathy affects the Ca^2+^-sensitivity, phosphorylation kinetics and proteolytic susceptibility of troponin. J Mol Cell Cardiol.

[100] Cheng Y., Rao V., Tu A.Y. (2015). Troponin I mutations R146G and R21C alter cardiac troponin function, contractile properties, and modulation by protein kinase A (PKA)-mediated phosphorylation. J Biol Chem.

[101] Wang Y., Pinto J.R., Solis R.S. (2012). Generation and functional characterization of knock-in mice harboring the cardiac troponin I-R21C mutation associated with hypertrophic cardiomyopathy. J Biol Chem.

[102] Psaras Y., Margara F., Cicconet M. (2021). CalTrack: high-throughput automated calcium transient analysis in cardiomyocytes. Circ Res.

[103] Biesiadecki B.J., Schneider K.L., Yu Z.B., Chong S.M., Jin J.P. (2004). An R111C polymorphism in wild Turkey cardiac troponin I accompanying the dilated cardiomyopathy-related abnormal splicing variant of cardiac troponin T with potentially compensatory effects. J Biol Chem.

[104] Wei H., Jin J.P. (2014). A dominantly negative mutation in cardiac troponin I at the interface with troponin T causes early remodeling in ventricular cardiomyocytes. Am J Physiol Cell Physiol.

[105] Barrick S.K., Greenberg L., Greenberg M.J. (2021). A troponin T variant linked with pediatric dilated cardiomyopathy reduces the coupling of thin filament activation to myosin and calcium binding. Mol Biol Cell.

[106] Dai Y., Amenov A., Ignatyeva N. (2020). Troponin destabilization impairs sarcomere-cytoskeleton interactions in iPSC-derived cardiomyocytes from dilated cardiomyopathy patients. Sci Rep.

[107] Luedde M., Ehlermann P., Weichenhan D. (2010). Severe familial left ventricular non-compaction cardiomyopathy due to a novel troponin T (TNNT2) mutation. Cardiovasc Res.

[108] Robinson P., Mirza M., Knott A. (2002). Alterations in thin filament regulation induced by a human cardiac troponin T mutant that causes dilated cardiomyopathy are distinct from those induced by troponin T mutants that cause hypertrophic cardiomyopathy. J Biol Chem.

[109] Schuldt M., Johnston J.R., He H. (2021). Mutation location of HCM-causing troponin T mutations defines the degree of myofilament dysfunction in human cardiomyocytes. J Mol Cell Cardiol.

[110] Hershberger R.E., Pinto J.R., Parks S.B. (2009). Clinical and functional characterization of TNNT2 mutations identified in patients with dilated cardiomyopathy. Circ Cardiovasc Genet.

[111] Li B., Guo Y., Zhan Y. (2021). Cardiac overexpression of XIN prevents dilated cardiomyopathy caused by *TNNT2* ΔK210 mutation. Front Cell Dev Biol.

[112] Bakkehaug J.P., Kildal A.B., Engstad E.T. (2015). Myosin activator omecamtiv mecarbil increases myocardial oxygen consumption and impairs cardiac efficiency mediated by resting myosin ATPase activity. Circ Heart Fail.

[113] Hartzell H.C., Glass D.B. (1984). Phosphorylation of purified cardiac muscle C-protein by purified cAMP-dependent and endogenous Ca^2+^-calmodulin-dependent protein kinases. J Biol Chem.

[114] Rittoo D., Jones A., Lecky B., Neithercut D. (2014). Elevation of cardiac troponin T, but not cardiac troponin I, in patients with neuromuscular diseases: implications for the diagnosis of myocardial infarction. J Am Coll Cardiol.

[115] Xu Z., Feng X., Dong J. (2017). Cardiac troponin T and fast skeletal muscle denervation in ageing. J Cachexia Sarcopenia Muscle.

[116] Shiraishi C., Matsumoto A., Ichihara K. (2023). RPL3L-containing ribosomes determine translation elongation dynamics required for cardiac function. Nat Commun.

[117] Rodriguez R., Harris M., Murphy E., Kennedy L.M. (2023). OGFOD1 modulates the transcriptional and proteomic landscapes to alter isoproterenol-induced hypertrophy susceptibility. J Mol Cell Cardiol.

[118] Stoehr A., Kennedy L., Yang Y. (2019). The ribosomal prolyl-hydroxylase OGFOD1 decreases during cardiac differentiation and modulates translation and splicing. JCI Insight.

[119] Zakhary D.R., Moravec C.S., Stewart R.W., Bond M. (1999). Protein kinase A (PKA)-dependent troponin-I phosphorylation and PKA regulatory subunits are decreased in human dilated cardiomyopathy. Circulation.

[120] Ward D.G., Cornes M.P., Trayer I.P. (2002). Structural consequences of cardiac troponin I phosphorylation. J Biol Chem.

[121] Biesiadecki B.J., Tachampa K., Yuan C., Jin J.P., de Tombe P.P., Solaro R.J. (2010). Removal of the cardiac troponin I N-terminal extension improves cardiac function in aged mice. J Biol Chem.

[122] van der Velden J., Papp Z., Zaremba R. (2003). Increased Ca^2+^-sensitivity of the contractile apparatus in end-stage human heart failure results from altered phosphorylation of contractile proteins. Cardiovasc Res.

[123] Belin R.J., Sumandea M.P., Sievert G.A. (2011). Interventricular differences in myofilament function in experimental congestive heart failure. Pflügers Arch Eur J Physiol..

[124] Sabater-Molina M., Pérez-Sánchez I., Hernández Del Rincón J.P., Gimeno J.R. (2018). Genetics of hypertrophic cardiomyopathy: a review of current state. Clin Genet.

[125] Poggesi C., Ho C.Y. (2014). Muscle dysfunction in hypertrophic cardiomyopathy: what is needed to move to translation?. J Muscle Res Cell Motil.

[126] Sewry C.A., Laitila J.M., Wallgren-Pettersson C. (2019). Nemaline myopathies: a current view. J Muscle Res Cell Motil.

[127] Tardiff J.C., Carrier L., Bers D.M. (2015). Targets for therapy in sarcomeric cardiomyopathies. Cardiovasc Res.

[128] Messer A.E., Marston S.B. (2014). Investigating the role of uncoupling of troponin I phosphorylation from changes in myofibrillar Ca^2+^-sensitivity in the pathogenesis of cardiomyopathy. Front Physiol.

[129] Papadaki M., Vikhorev P.G., Marston S.B., Messer A.E. (2015). Uncoupling of myofilament Ca^2+^ sensitivity from troponin I phosphorylation by mutations can be reversed by epigallocatechin-3-gallate. Cardiovasc Res.

[130] Keam S.J. (2022). Mavacamten: first approval [published correction appears in *Drugs*. 2022 Jul;82(11):1235]. Drugs.

[131] Harbowy M.E., Balentine D.A., Davies A.P., Cai Y. (1997). Tea chemistry. Crit Rev Plant Sci.

[132] Robinson P.J., Patel S., Liu X. (2016). Novel potential treatment of familial hypertrophic cardiomyopathy with analogues of the green tea polyphenol epigallocatechin-3-gallate. Biophys J.

[133] Tadano N., Du C.K., Yumoto F. (2010). Biological actions of green tea catechins on cardiac troponin C. Br J Pharmacol.

[134] Teerlink J.R., Malik F.I., Kass D.A. (2015). Letter by teerlink et al regarding article, “myosin activator omecamtiv mecarbil increases myocardial oxygen consumption and impairs cardiac efficiency mediated by resting myosin ATPase activity”. Circ Heart Fail.

[135] Li M.X., Robertson I.M., Sykes B.D. (2008). Interaction of cardiac troponin with cardiotonic drugs: a structural perspective. Biochem Biophys Res Commun.

[136] He H., Baka T., Balschi J. (2022). Novel small-molecule troponin activator increases cardiac contractile function without negative impact on energetics. Circ Heart Fail.

[137] Kimura A., Harada H., Park J.E. (1997). Mutations in the cardiac troponin I gene associated with hypertrophic cardiomyopathy. Nat Genet.

[138] Mogensen J., Kubo T., Duque M. (2003). Idiopathic restrictive cardiomyopathy is part of the clinical expression of cardiac troponin I mutations. J Clin Invest.

[139] Murphy R.T., Mogensen J., Shaw A., Kubo T., Hughes S., McKenna W.J. (2004). Novel mutation in cardiac troponin I in recessive idiopathic dilated cardiomyopathy. Lancet.

[140] Lu Q., Pan B., Bai H. (2021). Intranuclear cardiac troponin I plays a functional role in regulating *Atp2a2* expression in cardiomyocytes. Genes Dis.

[141] Wu H., Lee J., Vincent L.G. (2015). Epigenetic regulation of phosphodiesterases 2A and 3A underlies compromised β-adrenergic signaling in an iPSC model of dilated cardiomyopathy. Cell Stem Cell.

[142] Baryshnikova O.K., Li M.X., Sykes B.D. (2008). Modulation of cardiac troponin C function by the cardiac-specific N-terminus of troponin I: influence of PKA phosphorylation and involvement in cardiomyopathies. J Mol Biol.

[143] Landstrom A.P., Ackerman M.J. (2012). Beyond the cardiac myofilament: hypertrophic cardiomyopathy-associated mutations in genes that encode calcium-handling proteins. Curr Mol Med.

[144] Willott R.H., Gomes A.V., Chang A.N., Parvatiyar M.S., Pinto J.R., Potter J.D. (2010). Mutations in Troponin that cause HCM, DCM AND RCM: what can we learn about thin filament function?. J Mol Cell Cardiol.

[145] Davis J., Wen H., Edwards T., Metzger J.M. (2008). Allele and species dependent contractile defects by restrictive and hypertrophic cardiomyopathy-linked troponin I mutants. J Mol Cell Cardiol.

[146] Kapoor M., Das S., Biswas A. (2020). D190Y mutation in C-terminal tail region of TNNI_3_ gene causing severe form of restrictive cardiomyopathy with mild hypertrophy in an Indian patient. Meta Gene.

[147] Carballo S., Robinson P., Otway R. (2009). Identification and functional characterization of cardiac troponin I as a novel disease gene in autosomal dominant dilated cardiomyopathy. Circ Res.

[148] Hassoun R., Budde H., Mannherz H.G. (2021). *De novo missense mutations in TNNC1* and *TNNI*_*3*_ causing severe infantile cardiomyopathy affect myofilament structure and function and are modulated by troponin targeting agents. Int J Mol Sci.

[149] Dewan S., McCabe K.J., Regnier M., McCulloch A.D. (2017). Insights and challenges of multi-scale modeling of sarcomere mechanics in cTn and Tm DCM mutants-genotype to cellular phenotype. Front Physiol.

[150] Mirza M., Marston S., Willott R. (2005). Dilated cardiomyopathy mutations in three thin filament regulatory proteins result in a common functional phenotype. J Biol Chem.

[151] Van Acker H., De Sutter J., Vandekerckhove K., de Ravel T.J.L., Verhaaren H., De Backer J. (2010). Dilated cardiomyopathy caused by a novel TNNT2 mutation-added value of genetic testing in the correct identification of affected subjects. Int J Cardiol.

[152] Lu Q.W., Morimoto S., Harada K. (2003). Cardiac troponin T mutation R141W found in dilated cardiomyopathy stabilizes the troponin T-tropomyosin interaction and causes a Ca^2+^ desensitization. J Mol Cell Cardiol.

[153] Morales A., Pinto J.R., Siegfried J.D. (2010). Late onset sporadic dilated cardiomyopathy caused by a cardiac troponin T mutation. Clin Transl Sci.

[154] Pioner J.M., Vitale G., Gentile F. (2022). Genotype-driven pathogenesis of atrial fibrillation in hypertrophic cardiomyopathy: the case of different *TNNT2* mutations. Front Physiol.

[155] Hernandez O.M., Szczesna-Cordary D., Knollmann B.C. (2005). F110I and R278C troponin T mutations that cause familial hypertrophic cardiomyopathy affect muscle contraction in transgenic mice and reconstituted human cardiac fibers. J Biol Chem.

[156] Ezekian J.E., Clippinger S.R., Garcia J.M. (2020). Variant R94C in *TNNT2*-encoded troponin T predisposes to pediatric restrictive cardiomyopathy and sudden death through impaired thin filament relaxation resulting in myocardial diastolic dysfunction. J Am Heart Assoc.

[157] Wilkinson R., Song W., Smoktunowicz N., Marston S. (2015). A dilated cardiomyopathy mutation blunts adrenergic response and induces contractile dysfunction under chronic angiotensin II stress. Am J Physiol Heart Circ Physiol.

