# Supplementary material for: Focus on cardiac troponin complex: From gene expression to cardiomyopathy
Source: Genes Dis. 2024 Mar 11;11(6):101263. doi: 10.1016/j.gendis.2024.101263 (PMC11357864; doi:10.1016/j.gendis.2024.101263)
Supplement: Multimedia component 1 [file mmc1.docx]

**Supplementary material**

| **GENE/chromosome** | **RefSeqGene** | **Isoform**  **(adult/fetal)** | **Tissue** |
| --- | --- | --- | --- |
| **TNNC1**  **Chromosome**  **3p21.1** | NG_008963.1 | TnC (fetal/adult) | Heart/  Slow skeletal muscle |
| **TNNC2**  **Chromosome**  **20q13.12** | REVIEWED | fsTnC | Fast skeletal muscle |

**Supplemental Table 1. Gene encoding Troponin C (TnC) in cardiac and skeletal muscle**

fs: fast skeletal

**Supplemental Table 2. Gene encoding Troponin I (TnI) in cardiac and skeletal muscle**

| **GENE/chromosome** | **RefSeqGene** | **Isoform**  **(adult/fetal)** | **Tissue** |
| --- | --- | --- | --- |
| **TNNI3**  **Chromosome**  **19q13.42** | NG_007866.2 | cTnI (adult) | Heart |
| **TNNI1**  **Chromosome**  **1q32.1** | NG_016649.2 | ssTnI (fetal) |  |
| **TNNI1**  **Chromosome**  **1q32.1** | NG_016649.2 | ssTnI (fetal/adult) | Slow skeletal muscle |
| **TNNI2**  **Chromosome**  **11p15.5** | NG_011621.1 | fsTnI1 | Fast skeletal muscle |
|  |  | fsTnI2 |  |
|  |  | fsTnI3 |  |

c: cardiac; ss: slow skeletal; fs: fast skeletal.

| **GENE/chromosome** | **RefSeqGene** | **Isoform**  **(adult/fetal)** | **Tissue** |
| --- | --- | --- | --- |
| **TNNT2**  **chromosome 1q32.1** | NG_007556.1 | cTnT1 (fetal) | Heart |
|  |  | cTnT2(fetal) |  |
|  |  | cTnT3 (adult) |  |
|  |  | cTnT4 (fetal) |  |
| **TNNT1**  **chromosome 19q13.42** | NG_011829.2 | ssTnT a (adult) | Slow skeletal muscle |
|  |  | ssTnT b (adult) |  |
|  |  | ssTnT c (fetal) |  |
| **TNNT3**  **chromosome** **11p15.5** | NG_013085.2 | fsTnT3 (adult) | Fast skeletal muscle |
|  |  | fsTnT2 (fetal) |  |
|  |  | fsTnT4 (fetal) |  |
|  |  | fsTnT5 (fetal) |  |
|  |  | fsTnT6 (adult) |  |
|  |  | fsTnT7 |  |
|  |  | fsTnT8 |  |
|  |  | fsTnT9 |  |
|  |  | fsTnT10 |  |
|  |  | fsTnT11 |  |
|  |  | fsTnT12 (fetal) |  |
|  |  | fsTnT1(adult) |  |

**Supplemental Table 3. Gene encoding Troponin T (TnT) in cardiac and skeletal muscle.**

c: cardiac; ss: slow skeletal; fs: fast skeletal.

**Supplemental Table 4. Comparison between Tns subunits of cardiac muscles (cTns) with those of striated skeletal muscles (ssTns and fsTns).**

| **Tns** | **Protein alignment** |
| --- | --- |
| **TnC** | TnC MDDIYKAAVEQLTEEQKNEFKAAFDIFVLGAEDGCISTKELGKVMRMLGQNPTPEELQEM 60  fsTnC MTDQQAEARSYLSEEMIAEFKAAFDMFDAD-GGGDISVKELGTVMRMLGQTPTKEELDAI 59  * * * * ** ******* * * ** **** ******* ** ***  TnC IDEVDEDGSGTVDFDEFLVMMVRCMKDDSKGKSEEELSDLFRMFDKNADGYIDLDELKIM 120  fsTnC IEEVDEDGSGTIDFEEFLVMMVRQMKEDAKGKSEEELAECFRIFDRNADGYIDPEELAEI 119  * ********* ** ******** ** * ******** ** ** ******* **  TnC LQATGETITEDDIEELMKDGDKNNDGRIDYDEFLEFMKGVE 161  fsTnC FRASGEHVTDEEIESLMKDGDKNNDGRIDFDEFLKMMEGVQ 160  * ** * ** ************** **** * ** |
| **TnI** | cTnI MADGSSDAAREPRPAPAPIRRRSSNYRAYATEPHAKKKSKISASRKLQLKTLLLQIAKQE 60  ssTnI -------------------------------MPEVERKPKITASRKLLLKSLMLAKAKEC 29  fsTnI1 -------------------------------MGDEEKRNRAITARRQHLKSVMLQIAATE 29  * ** * *  cTnI LEREAEERRGEKGRALSTRCQPLELAGLGFAELQDLCRQLHARVDKVDEERYDIEAKVTK 120  ssTnI WEQEHEEREAEKVRYLAERIPTLQTRGLSLSALQDLCRELHAKVEVVDEERYDIEAKCLH 89  fsTnI1 LEKEESRREAEKQNYLAEHCPPLHIPG-SMSEVQELCKQLHAKIDAAEEEKYDMEVRVQK 88  * * * ** * * * * ** *** ** ** *  cTnI NITEIADLTQKIFDLRGKFKRPTLRRVRISADAMMQALLGARAKESLDLRAHLKQVKKED 180  ssTnI NTREIKDLKLKVMDLRGKFKRPPLRRVRVSADAMLRALLGSKHKVSMDLRANLKSVKKED 149  fsTnI1 TSKELEDMNQKLFDLRGKFKRPPLRRVRMSADAMLKALLGSKHKVCMDLRANLKQVKKED 148  * * * ********* ***** ***** **** * **** ** *****  cTnI TEKEN--REVGDWRKNIDALSGMEGRKKKFES------- 210  ssTnI TEKERP-VEVGDWRKNVEAMSGMEGRKKMFDAAKSPTSQ 187  fsTnI1 TEKERDLRDVGDWRKNIEEKSGMEGRKKMFESES----- 182  **** ******* ******** * |
| **TnT** | cTnT3 MSDIEEVVEEYEEEEQEEAAVEEQEEAAEEDAEAEAETEETRAEEDEEEEEAKEAEDGPM 60  ssTnT_a MSDTEE--QEYEEEQPEEEAA----------------------EEEEEAPEEPEPVAEPE 36  ssTnT_b MSDTEE--QEYEEEQPEEEAA----------------------EEEEEAPEEPEPVAEPE 36  fsTnT1 MSDEEV--EQVEEQYEEEEEA----------------------QEEEEVQED-TAEEDAE 35  fsTnT3 MSDEEV--EQVEEQYEEEEEA----------------------QEEE---------EVQE 27  fsTnT6 MSDEEV--EQVEEQYEEEEEA----------------------QEEAAEV---HEEVHEP 33  *** * ** ** *  cTnT3 EESKPKPRSFMPNLVPPKIPDGERVDFDDIHRKRMEKDLNELQALIEAHFENRKKEEEEL 120  ssTnT_a EERPKPSRPVVPPLIPPKIPEGERVDFDDIHRKRMEKDLLELQTLIDVHFEQRKKEEEEL 96  ssTnT_b EERPKPSRPVVPPLIPPKIPEGERVDFDDIHRKRMEKDLLELQTLIDVHFEQRKKEEEEL 96  fsTnT1 E------EKPRPKLTAPKIPEGEKVDFDDIQKKRQNKDLMELQALIDSHFEARKKEEEEL 89  fsTnT3 E------EKPRPKLTAPKIPEGEKVDFDDIQKKRQNKDLMELQALIDSHFEARKKEEEEL 81  fsTnT6 E------EKPRPKLTAPKIPEGEKVDFDDIQKKRQNKDLMELQALIDSHFEARKKEEEEL 87  * * * **** ** ****** ** *** *** ** *** ********  cTnT3 VSLKDRIERRRAERAEQQRIRNEREKERQNRLAEERARREEEENRRKAEDEARKKKALSN 180  ssTnT_a VALKERIERRRSERAEQQRFRTEKERERQAKLAEEKMRKEEEEAKKRAEDDAKKKKVLSN 156  ssTnT_b VALKERIERRRSERAEQQRFRTEKERERQAKLAEEKMRKEEEEAKKRAEDDAKKKKVLSN 156  fsTnT1 VALKERIEKRRAERAEQQRIRAEKERERQNRLAEEKARREEEDAKRRAEDDLKKKKALSS 149  fsTnT3 VALKERIEKRRAERAEQQRIRAEKERERQNRLAEEKARREEEDAKRRAEDDLKKKKALSS 141  fsTnT6 VALKERIEKRRAERAEQQRIRAEKERERQNRLAEEKARREEEDAKRRAEDDLKKKKALSS 147  * ** *** ** ******* * * * *** **** * *** *** *** **  cTnT3 M-MHFGGYIQKQAQTERKSGKRQTEREKKKKILAERRKVLAIDHLNEDQLREK------- 232  ssTnT_a MGAHFGGYLVKA---EQKRGKRQTGREMKVRILSERKKPLDIDYMGEEQLRARSAWLPPS 213  ssTnT_b MGAHFGGYLVKA---EQKRGKRQTGREMKVRILSERKKPLDIDYMGEEQLR--------- 204  fsTnT1 MGANYSSYLAKA---DQKRGKKQTAREMKKKILAERRKPLNIDHLGEDKLRDK------- 199  fsTnT3 MGANYSSYLAKA---DQKRGKKQTAREMKKKILAERRKPLNIDHLGEDKLRDK------- 191  fsTnT6 MGANYSSYLAKA---DQKRGKKQTAREMKKKILAERRKPLNIDHLGEDKLRDK------- 197  * * * * ** ** ** * ** ** * * ** * ** |
| **TnT** | cTnT3 ---------AKELWQSIYNLEAEKFDLQEKFKQQKYEINVLRNRINDNQKVSKTR---GK 280  ssTnT_a QPSCPAREKAQELSDWIHQLESEKFDLMAKLKQQKYEINVLYNRISHAQKFRKGA---GK 270  ssTnT_b -------EKAQELSDWIHQLESEKFDLMAKLKQQKYEINVLYNRISHAQKFRKGA---GK 254  fsTnT1 ---------AKELWETLHQLEIDKFEFGEKLKRQKYDITTLRSRIDQAQKHSKKAGTPAK 250  fsTnT3 ---------AKELWETLHQLEIDKFEFGEKLKRQKYDIMNVRARVQMLAKFSKKAGTPAK 242  fsTnT6 ---------AKELWETLHQLEIDKFEFGEKLKRQKYDIMNVRARVQMLAKFSKKAGTPAK 248  * ** ** ** * * *** * * * * *  cTnT3 AKVTGRWK 288  ssTnT_a GRVGGRWK 278  ssTnT_b GRVGGRWK 262  fsTnT1 GKVGGRWK 258  fsTnT3 GKVGGRWK 250  fsTnT6 GKVGGRWK 256  * **** |

Aminoacids specific for cTns are highlighted in red and those in common with skeletal Tns are marked with *.
